# Insights into the adsorption and corrosion inhibition properties of newly synthesized diazinyl derivatives for mild steel in hydrochloric acid: synthesis, electrochemical, SRB biological resistivity and quantum chemical calculations

**DOI:** 10.1039/d2ra06574f

**Published:** 2022-12-22

**Authors:** Mahmoud A. Bedair, Hani M. Elaryian, Ehab S. Gad, Mubark Alshareef, Ahmed H. Bedair, Rabab M. Aboushahba, Abd El-Aziz S. Fouda

**Affiliations:** Department of Chemistry, Faculty of Science (Men's Campus), Al-Azhar University Nasr City 11884 Cairo Egypt m_bedier@azhar.edu.eg m_bedier@yahoo.com; College of Science and Arts, University of Bisha P.O. Box 101 Al-Namas 61977 Saudi Arabia mbedair@ub.edu.sa; Zohr Gas Field, Belayim Petroleum Company Nasr City 7074 Cairo Egypt; Chemistry Department, College of Science and Arts, Jouf University Saudi Arabia; Department of Chemistry, Faculty of Applied Science, Umm Al Qura University Makkah 24230 Saudi Arabia mmshreef@uqu.edu.sa; Department of Chemistry, Faculty of Science (Girl's Branch), Al-Azhar University Nasr City 11574 Cairo Egypt; Department of Chemistry, Faculty of Science, Mansoura University Mansoura-35516 Egypt asfouda@hotmail.com

## Abstract

Two azo derivatives, 4-((4-hydroxy-3-((4-oxo-2-thioxothiazolidin-5-ylidene)methyl)phenyl) diazinyl) benzenesulfonic acid (TODB) and 4-((3-((4,4-dimethyl-2,6-dioxocyclohexylidene) methyl)-4-hydroxyphenyl)diazinyl) benzenesulfonic acid (DODB) were synthesized and characterized using Fourier-transform infrared spectroscopy (FTIR), proton nuclear magnetic resonance (^1^H-NMR) and mass spectral studies. Gravimetric methods, potentiodynamic polarization (PDP), electrochemical impedance spectroscopy (EIS), electrochemical frequency modulation (EFM) techniques and inductive coupled plasma-optical emission spectroscopy were used to verify the above two compounds' ability to operate as mild steel (MS) corrosion inhibitors in 1 M HCl. Tafel data suggest that TODB and DODB have mixed-type characteristics, and EIS findings demonstrate that increasing their concentration not only alters the charge transfer (*R*_ct_) of mild steel from 6.88 Ω cm^2^ to 112.9 Ω cm^2^ but also changes the capacitance of the adsorbed double layer (*C*_dl_) from 225.36 to 348.36 μF cm^−2^. At 7.5 × 10^−4^ M concentration, the azo derivatives showed the highest corrosion inhibition of 94.9% and 93.6%. The inhibitory molecule adsorption on the metal substrate followed the Langmuir isotherm. The thermodynamic activation functions of the dissolution process were also calculated as a function of inhibitor concentration. UV-vis, scanning electron microscopy (SEM), and energy dispersive X-ray spectroscopy (EDX) techniques were used to confirm the adsorption phenomenon. The quantum chemical parameters, inductively coupled plasma atomic emission spectroscopy (ICPE) measurements, and the anti-bacterial effect of these new derivatives against sulfate-reducing bacteria (SRB) were also investigated. Taken together, the acquired results demonstrate that these compounds can create an appropriate preventing surface and regulate the corrosion rate.

## Abstract 2

Two azo derivatives (TODB) and (DODB) were synthesized and characterized as mild steel (MS) corrosion inhibitors in 1 M HCl. Electrochemical data suggest that TODB and DODB are excellent inhibitors. They show anti-bacterial effect against SRB.

## Graphical abstract

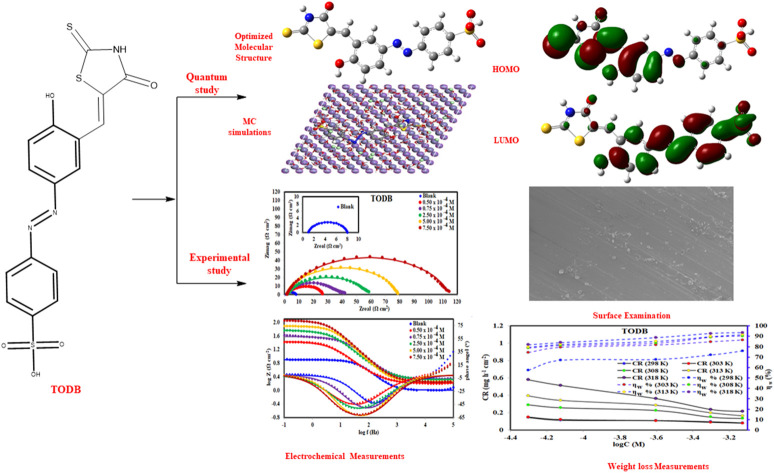

## 1. Introduction

Mild steel (MS) is a material with small carbon content that is solid and tough but not easily tempered. It is known as low-carbon steel, and it is currently the most well-known type of steel because of its relatively low cost while providing mechanical characteristics that are sufficient for certain implementations.^1,2^ Mineral acids, especially corrosive hydrochloric acid, are regularly utilized for mechanical purposes, such as corrosive cleaning, corrosive pickling, corrosive descaling, and oil well acidizing. Such acids create highly corrosive surroundings for MS. As a direct consequence, the study of steel protection against corrosion is always a topic of great theoretical and practical importance. Moreover, because MS is susceptible to corrosion like other metallic materials, its surface must be protected from such undesirable process. Corrosion inhibitors have recently been used to eliminate/reduce corrosion of metal parts in home appliances and industrial machines. Aside from the use of traditional inhibitors,^3,4^ metals can be also protected through chemical or electrochemical surface functionalization, such as SAMs (Self-assembled Monolayers) formed from silanes,^5,6^ phosphonic acids,^7^ or electrochemical reduction of aryldiazonium salts on metals.^8,9^ Organic compounds with π-electron systems, atoms with lone pair electrons (P, S, N, O), and plane-conjugated structures including benzene rings are the most effective organic inhibitors.^10–12^ The electrical connections between the organic inhibitors and the metal surface are facilitated by certain sets of atoms or bonds, which allows the inhibitors to adhere to the base metal. It has been asserted that the efficiency of these molecules for corrosion protection increases in the following order: O > N > S > P.^8,13^ In most cases, the efficiencies of these organic inhibitors are determined by the components of the environment in which they act, such as the nature of the metal substrate, the electrochemical potential at the metal/solution interface, the metal surface protected area, the inhibitor's molecular size, manner of adsorption, its concentration, and its structure.^14^ The choice of any inhibitor, on the other hand, is heavily influenced by its economic allocation, efficiency in inhibiting the substrate material, and environmental side effects.^15^ In general, organic dyes have especially gained significant attention due to their multipurpose applications in a variety of fields, such as cosmetics, textiles, food, and pharmaceuticals.^16^ Because of their distinct chemical structures, they are also used in the protection of metals and alloys against corrosion. Azo-dyes have shown significant inhibition efficiencies^17,18^ and are one of the most commonly used organic compounds as inhibitors. These compounds' inhibition process has been shown to implement an inhibitor adsorption isotherm,^17,18^ whereas their efficiency is dependent on the structure and chemical properties of the adsorbed inhibitor film formed on the steel surface. Density functional theory (DFT) has become a helpful strategy in deriving the electronic properties, allowing researchers to obtain solid basic parameters for the atoms.^19,20^ In corrosion studies, this strategy makes it conceivable to precisely anticipate the hindrance productivity of natural erosion inhibitors on the premise of the electronic and atomic properties, as well as the reactivity records. Many studies have been conducted and published previously on the use of conceptual models for predicting and supporting the corrosion inhibitive potentials of molecules, including azo dyes.^21–23^ In light of this, two azo derivatives, TODB and DODB compounds were evaluated as novel inhibitors of MS corrosion in a corrosive media of HCl (1.0 M) solution. TODB and DODB compounds were thoroughly characterized using FTIR, proton nuclear magnetic resonance (^1^H-NMR), and mass spectral studies. At a constant temperature, various techniques such as PDP, EIS, and SEM were used to assess the inhibition efficiencies (% IEs) of the examined organic molecules.

## 2. Experimental

### 2.1. Synthesis of TODB and DODB compounds

**Fig. 1:**
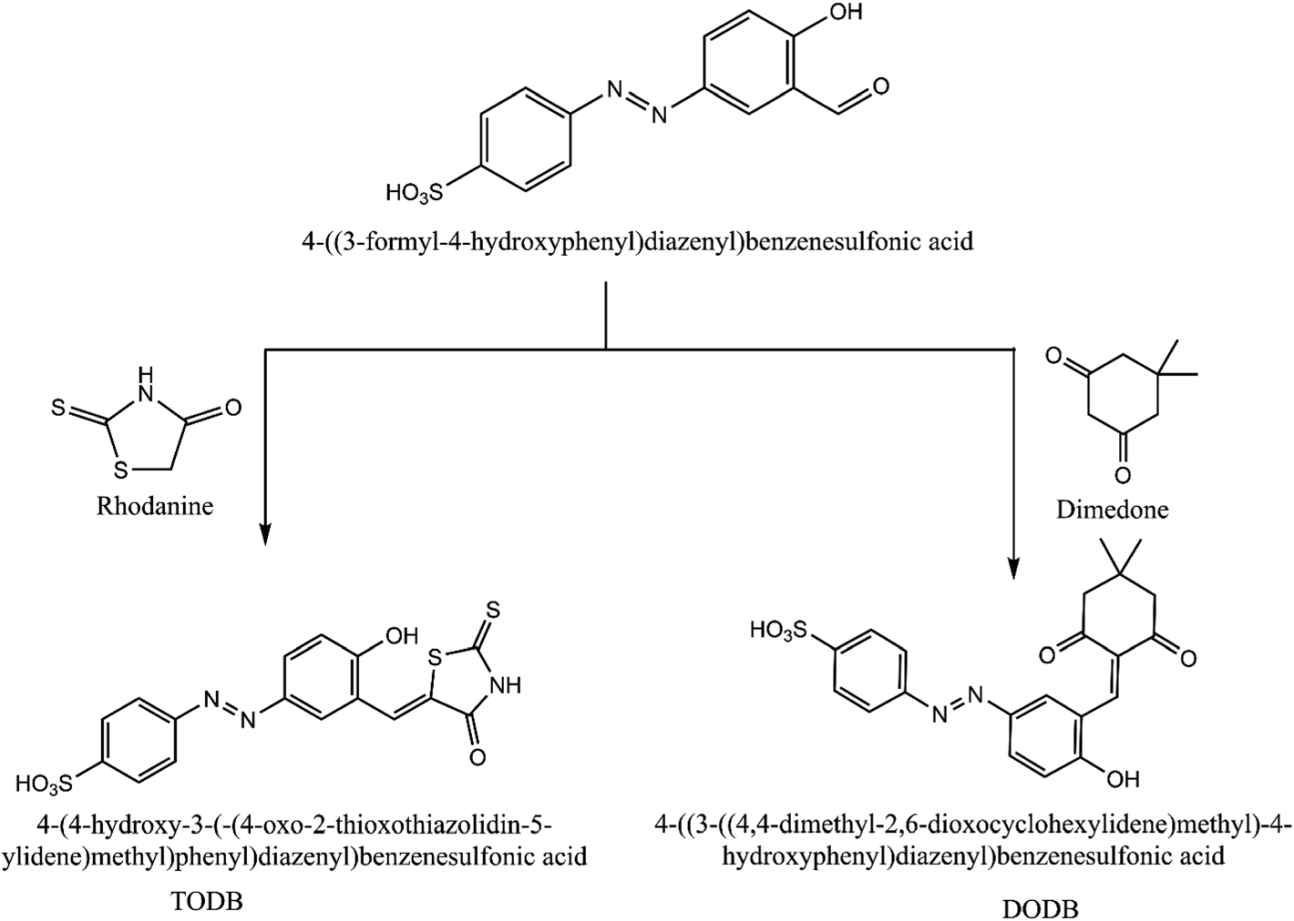
Scheme for synthesized inhibitors TODB and DODB.

4-((3-Formyl-4-hydroxyphenyl) diazenyl) benzenesulfonic acid (0.013 mol) was prepared and mixed with two cyclic ketones, rhodanine (0.013 mol) and dimedone (0.021 mol), in a round bottom flask with a few drops of piperidine, acetic acid and ethanol as the solvent (100 ml). The reaction mixture was refluxed with stirring for 2 h, and filtered to omit the solvent. The recrystallization of the precipitate was performed using ethanol, then left to dry to give the final product (Fig. 1). TODB; brown powder, yield: 90.84%, mp: over 300 °C. DODB; yellow powder, low yield: 15.43%, mp: over 300 °C.

### 2.2. Corrosive environment

One molar HCl stock solution was made by dilution with double-distilled water. Furthermore, the concentration varieties of the studied compounds were (0.50 × 10^−4^–7.50 × 10^−4^) and were prepared using ethanol.

### 2.3. Gravimetric measurements

Weight loss (WL) tests were carried by inserting the tested sheets into an aerated acidic solution HCl (0.1 M) with and without various concentrations of the prepared TODB and DODB at warm temperatures from 298 to 318 K. After a 6 hour soaking, the samples were immediately removed, washed, dried, and weighed. The assessed weight loss was used to quantify the degree of surface coverage (*θ*) and the inhibition efficiency (% IE) using eqn (1) and (2):^24^

1 *θ* = (*W*_o_ − *W*_inh_)/*W*_o_

2 % IE = [(*W*_o_ − *W*_inh_)/ *W*_o_] × 100

where *W*_o_ and *W*_inh_ are the weight loss values (mg) without and with inhibitors. Using eqn (3), the corrosion rate, *C*_R_ (mg cm^−2^ h^−1^) was assessed;^25^

3 *C*_R_ = *W*/(*S* × *t*)

where *S* is the surface area of the metal samples (cm^2^), and *t* is the exposure time (h).

### 2.4. Electrochemical measurements

For studying the inhibition of MS corrosion using TODB and DODB, three distinct electrochemical techniques were used: PDP, EIS, and electrochemical frequency modulation (EFM). At 25 °C, a standard electrochemical cell fabricated from MS (1 cm^2^ exposed area), a saturated calomel electrode (SCE) and a Pt sheet as the working, reference, and counter electrodes, respectively, was used for electrochemical studies.

#### 2.4.1. PDP technique

Utilizing Gamry framework devices, potentiodynamic current–potential graphs were documented by instantaneously shifting the electrode potential from −1500 mV to +500 mV at a scanning rate of 1 mV s^−1^ (version 3.20). Corrosion current densities (*I*_corr_) and corrosion potential (*E*_corr_) were assessed from the interplay of the correlation anodic and cathodic sections of Tafel plots in the absence and presence of various inhibitor concentrations. Using the degree of surface coverage (*θ*) from eqn (4), the percentage inhibition efficiency (percent IE) was quantified:^4,26^

4 % IE = *θ* × 100 = [1 − (*I*_corr_/*I*^o^_corr_)] × 100

where *I*^o^_corr_ and *I*_corr_ are the corrosion current densities without and with TODB and DODB derivatives, respectively.

#### 2.4.2. EIS measurements

After immersing the electrode for 15 minutes, the EIS spectra were collected at the open circuit potential, OCP. The peak-to-peak voltage of the AC signal was 10 mV, and the resonant frequency evaluated was 0.1–10^5^ Hz. Nyquist and Bode plots were used to present the results. The important variables derived from the analysis of the Nyquist diagram are the resistance of charge transfer (*R*_ct_) and the capacity of the double layer (*C*_dl_), which are estimated using eqn (5):^27^5
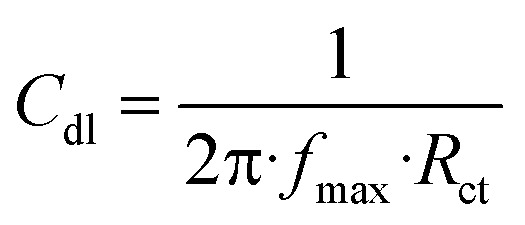
where *f*_max_ is the maximum frequency. The terms (*θ*) and (% IE) were also estimated using eqn (6):^28^

6 %IE = *θ* × 100 = [1 − (*R*^o^_ct_/*R*_ct_)]

where *R*^o^_ct_ and *R*_ct_ are the charge transfer resistances without and in presence of different concentrations of TODB and DODB, respectively.

#### 2.4.3. EFM technique

EFM was used with 2 and 5 Hz frequencies. Because the reference frequency was 1 Hz, the waveform repeated after 1 second. The higher frequency should be slow enough that the charging of the double layer does not make a contribution to the current approach. Before initiating the readings, the electrode potential was stabilized for 30 minutes. Gamry reference 3000 Potentiostat/Galvanostat/ZRA analyzer, DC 105 corrosion, EIS 300, EFM 140, and Echem Analyst 5.21 software were used for data plotting, graphing, data fitting, and calculating.

### 2.5. ICPE technique

In this research, the content of dissolved MS in HCl (1.0 M) solutions with and without various amounts of TODB and DODB was determined by ICPE. The ICPE-9820 from SHIMADZU used for our studies was standardized using 1, 5, and 10 ppm made from a 1000 ppm Scharlau iron standard stock solution. The MS coupons were submerged in various doses of the newly synthesized compounds for 24 hours. The content of dissolved iron was then measured in the solutions after dilution with ultra-pure demineralized water to obtain results that were plotted on the calibration graph for the instrument.^29^

### 2.6. UV-analysis

An ultraviolet-visible spectrophotometer (Thermo Fisher Scientific) was employed to examine the shift in wavelength following MS immersion in HCl (1.0 M) for 24 h, both with and without the addition of TODB and DODB. A shift in wavelength was a strong indicator that a combination between the inhibitor molecules and the Fe^2+^ on the electrode had formed.^30^

### 2.7. SEM and EDX tests

The MS surface was studied using the SEM JEOL JSM-IT200 apparatus with and without the application of an appropriate molarity of TODB and DODB after being submerged in HCl (1.0 M) for one day. The quantitative concentration of the constituents from the solutions adsorbed on the MS surface was also determined using EDX.

### 2.8. Biological activity

H_2_S is produced by a kind of anaerobic bacteria (SRB), which can speed up the corrosion process. The test was conducted using SRB-BART™ testers from Droycon Bioconcepts, Inc. (DBI). The test time is 11 days, and the test results can be obtained by counting the number of bacterial colonies present when the test vials first turn black. Each day refers to a certain quantity of germs that are present.^31^

### 2.9. Theoretical chemical parameters

Using Gaussian 09 with Gauss View 06 software with different basis sets (semi-empirical PM6, Hartree–Fock 631G, DFT 6311G and MP2-6-311G), the optimized chemical calculations were performed. Different parameters were obtained *via* the calculations. The highest (*E*_HOMO_) occupied and lowest (*E*_LUMO_) unoccupied molecular orbitals energies, and the energy gap (Δ*E*) between them were calculated. Other parameters were also optimized, including IP (ionization potential), EA (electron affinity), *χ* (electronegativity), *ω* (electrophilicity), Δ*N* (transferred electrons), *σ* (softness), *η* (hardness), μ (dipole moment), MV (molecular volume) and TNC (total negative charge). According to the following equations, the different parameters can be calculated as follows:^32–35^

7 Δ*E* = *E*_LUMO_ − *E*_HOMO_

8 *E*_LUMO_ = −EA

9 *E*_HOMO_ = −IP

10 *η* = −(EA − IP)/2

11 *χ* = (EA + IP)/2

12 *σ* = 1/*η*

13
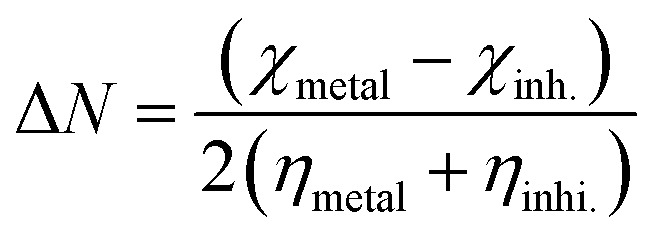


The interactions between the TODB and DODB molecules (either in neutral or protonated phase) and the mild steel surface were theoretically investigated by Monte Carlo simulations using adsorption locator module in Material studio 2017 software. The acidic aqueous conditions were brought by adding 200 H_2_O, 20 H_3_O^+^ and Cl^−^. Fe was cleaved over the most stable (110) plane. The Fe (110) was expanded to a (10 × 10) super cell, and a vacuum with 30 Å was formed above the Fe (110) plane. To find the equilibrium adsorption configurations, the COMPASS force field was used.

## 3. Results and discussions

### 3.1. Structure confirmation of the prepared compounds

#### 3.1.1. FTIR spectrum (KBr, *ν*_max_, cm^−1^)

**Fig. 2:**
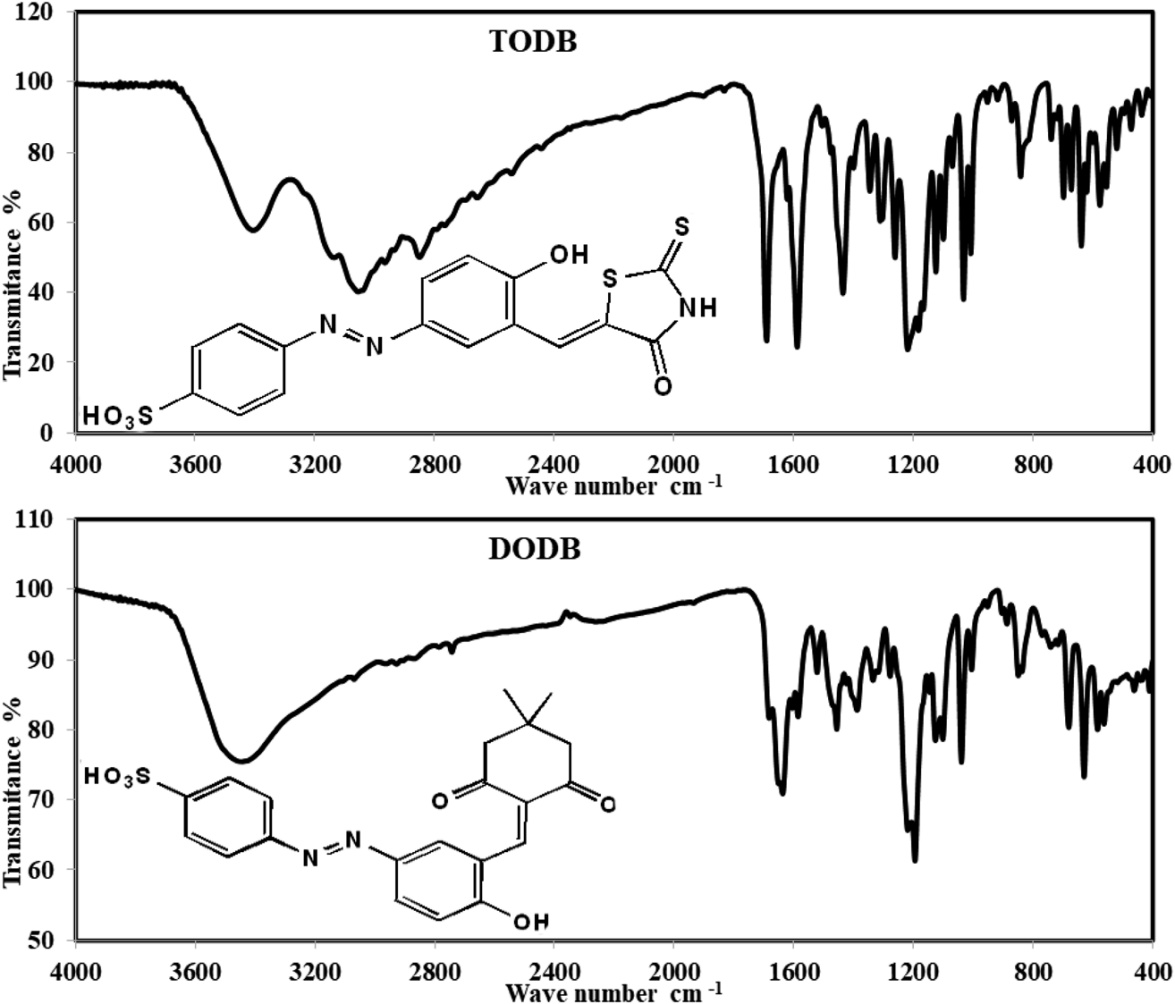
FTIR spectrum for synthesized inhibitors TODB and DODB.

The IR spectrum revealed characteristic bands of the two synthesized compounds, as shown in Fig. 2. The infrared spectrum of TODB revealed clear absorption bands at 2849.04 and 2964.92 cm^−1^ related to the bending vibration of the (Aliphatic C–H) group. The band at 3054.29 cm^−1^ is attributed to the stretching vibration of the (Aromatic C–H). The bands at 3137.68 and 1620.01 cm^−1^ refer to the stretching vibration of (NH). The band at 1433.65 cm^−1^ is attributed to the stretching vibration of the azo group (N=N). The band at 1688.74 cm^−1^ is attributed to the stretching vibration of (C=O). The band at 1620.01 cm^−1^ is attributed to the stretching vibration of *ν* (C=C) arylidene group and overlapped with (C=O) at 1688.74 cm^−1^. The band at 1030.84 cm^−1^ is attributed to the stretching vibration of (C=S). Finally, the band at 3404.59 cm^−1^ is attributed to the (Phenolic OH, SO_3_H) groups.

On the other hand, the infrared spectrum of DODB revealed clear absorption bands at 2742.55, 2866.72, 2931.48 cm^−1^, which were attributed to the bending vibration of the (Aliphatic C–H) group. The band at 3066.88 cm^−1^ owing to the stretching vibration of (Aromatic C–H), and the bands at 1637.73 cm^−1^ referred to the stretching vibration of the (C=O) ketone. The band at 1457.45 cm^−1^ is attributed to the stretching vibration of the azo group (N=N). The band at 1683.41 cm^−1^ is attributed to the stretching vibration of *ν* (C=C) arylidene group and overlapped with (C=O) at 1637.73 cm^−1^. Finally, the band at 3448.31 cm^−1^ is attributed to (Phenolic OH, SO_3_H) groups.

The frequency calculations were performed on the TODB and DODB compounds using DFT/B3LYP/6-311+G and HF-631G basis sets. The calculated frequencies and scale factors are listed in Table 1. The calculated data were compatible with the experimental frequencies, as the scale factor values were close to unity.

**Table 1:** Frequencies and scale factor of the IR-active vibrational modes of TODB and DODB resulting from DFT, HF calculations and experiment

Assignment	TODB (IR frequencies cm^−1^)					DODB (IR frequencies cm^−1^)				
	Experimental	Scale factor	DFT/B3LYP/6-311+G	Scale factor	HF-631G	Experimental	Scale factor	DFT/B3LYP/6-311+G	Scale factor	HF-631G
Phenolic OH	3404.59	0.9190	3704.73	0.8839	3851.88	3448.31	0.9317	3701.22	0.8477	4068.08
NH	3137.68	0.8713	3601.05	0.8259	3799.26	—	—	—	—	—
Aromatic CH	3054.29	0.9516	3209.48	0.8977	3402.37	3066.88	0.9556	3209.30	0.9007	3404.91
Aliphatic CH	2964.92	0.9510	3117.71	0.8730	3396.27	2931.48	0.9505	3084.10	0.9236	3173.92
C=O	1688.74	0.9999	1688.86	0.9833	1717.44	1683.41	1.0029	1678.58	0.9104	1849.08
N=N	1433.65	0.9720	1475.01	0.9968	1438.23	1457.45	0.9860	1478.21	0.8309	1753.96
C=S	1030.84	1.0043	1026.46	0.9120	1130.30	—	—	—	—	—

#### 3.1.2. ^1^H_0_ NMR (DMSO-*d*_6_)

**Fig. 3:**
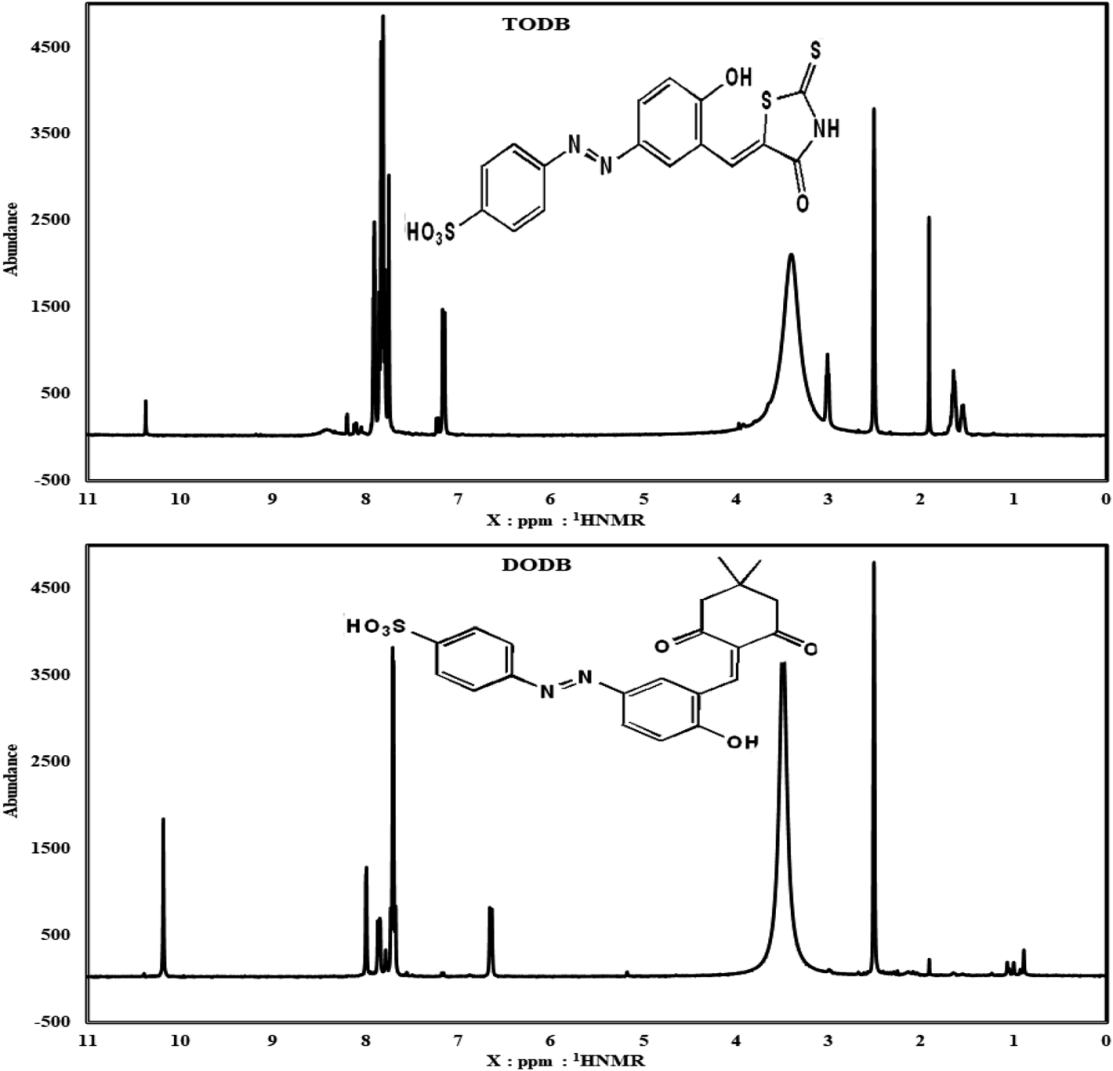
HNMR spectrum for synthesized inhibitors TODB and DODB.

Fig. 3 shows the ^1^HNMR for TODB, which is 400 MHz; at: *δ* = 3.1 ppm (1H, s, NH), 7.14 ppm (1H, s, HC=C) (Arylidene), 1.91 ppm (1H, s, Phenolic OH), *δ* = 10.37 ppm (SO_3_H)), *δ* = 7.9–8.20 ppm (7H, multiplet, Ar–H). ^1^HNMR for DODB is 400 MHz; at: *δ* = 7.18, 7.15 (1H, d, *J* = 12 Hz, HC=C) (Arylidene), *δ* = 1.91 ppm (Phenolic OH), *δ* = 10.39 ppm (SO_3_H), *δ* = 7.86–7.78 ppm (7H, multiplet, Ar–H), *δ* = 5.17 ppm ((1H,s, –OH=CH–(CH_3_)_2_), *δ* = 0.89, 1.0 ppm ((CH_3_)_2_).

#### 3.1.3. Mass spectrum

**Fig. 4:**
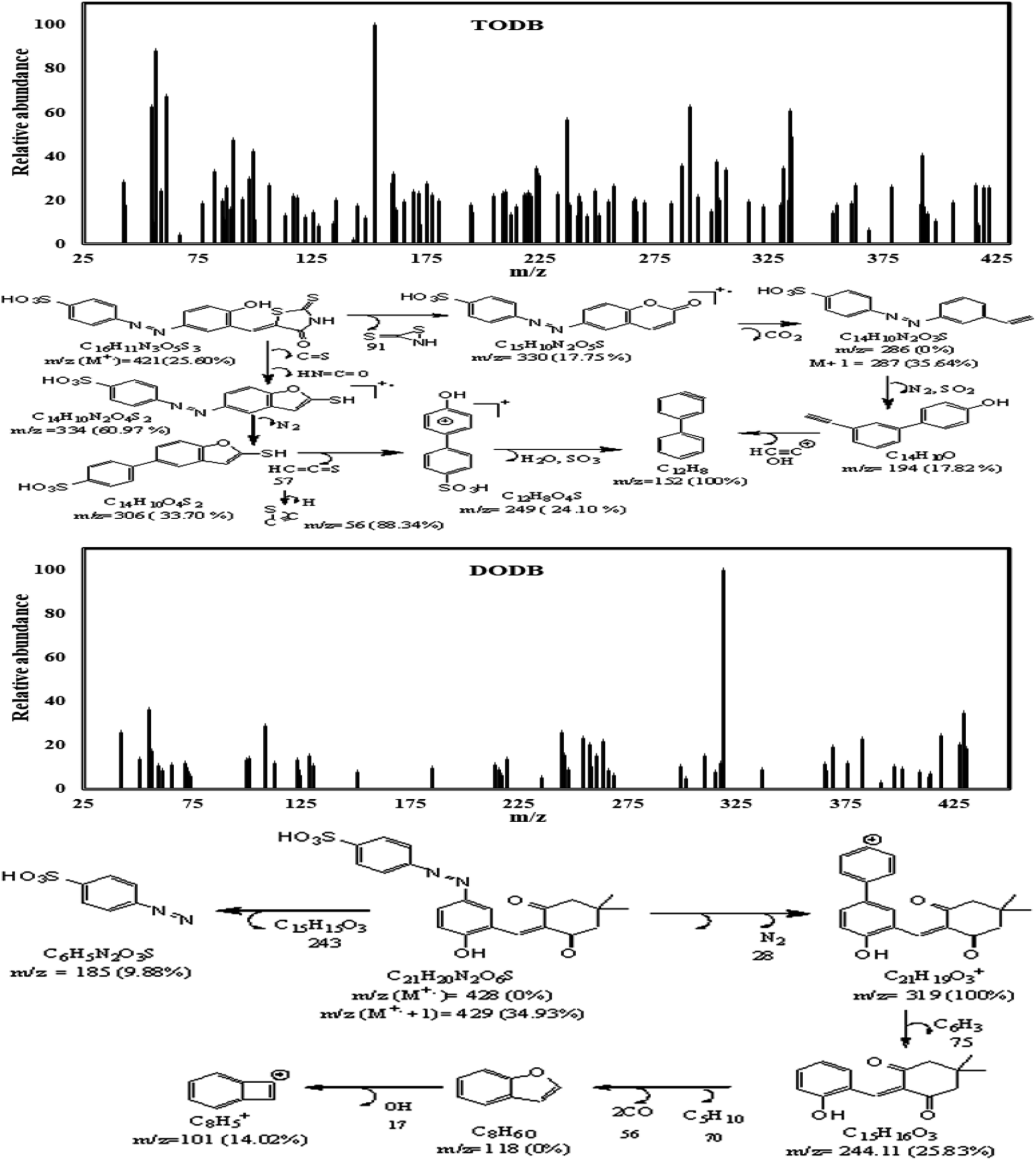
Mass spectrum and fragmentations for synthesized inhibitors TODB and DODB.

The mass spectrum (Fig. 4) for TODB showed that *m*/*z* (M^+.^) = 421 (25.60%) and the molecular ion peak as the base peak at *m*/*z* (%); 152 (100%) together with other peaks at *m*/*z* (%); 330 (17.75%), 249 (24.10%), 56 (88.34%), 306 (33.70%), 334 (60.97%), 286 (0%), M + 1 = 287 (35.64%) &194 (17.82%). The mass spectrum for DODB (Fig. 4) showed that the *m*/*z* (M+.) = 428 (0%), (M+. +1) = 429 (34.93%), (M+. −1) = 427 (20.39%) and molecular ion peak as the base peak at *m*/*z* (%); 319 (100%) together with other peaks at *m*/*z* (%); 244 (25.83%), 185 (9.88%) &101 (14.02%).

### 3.2. Evaluation techniques

#### 3.2.1. Gravimetric results

**Fig. 5:**
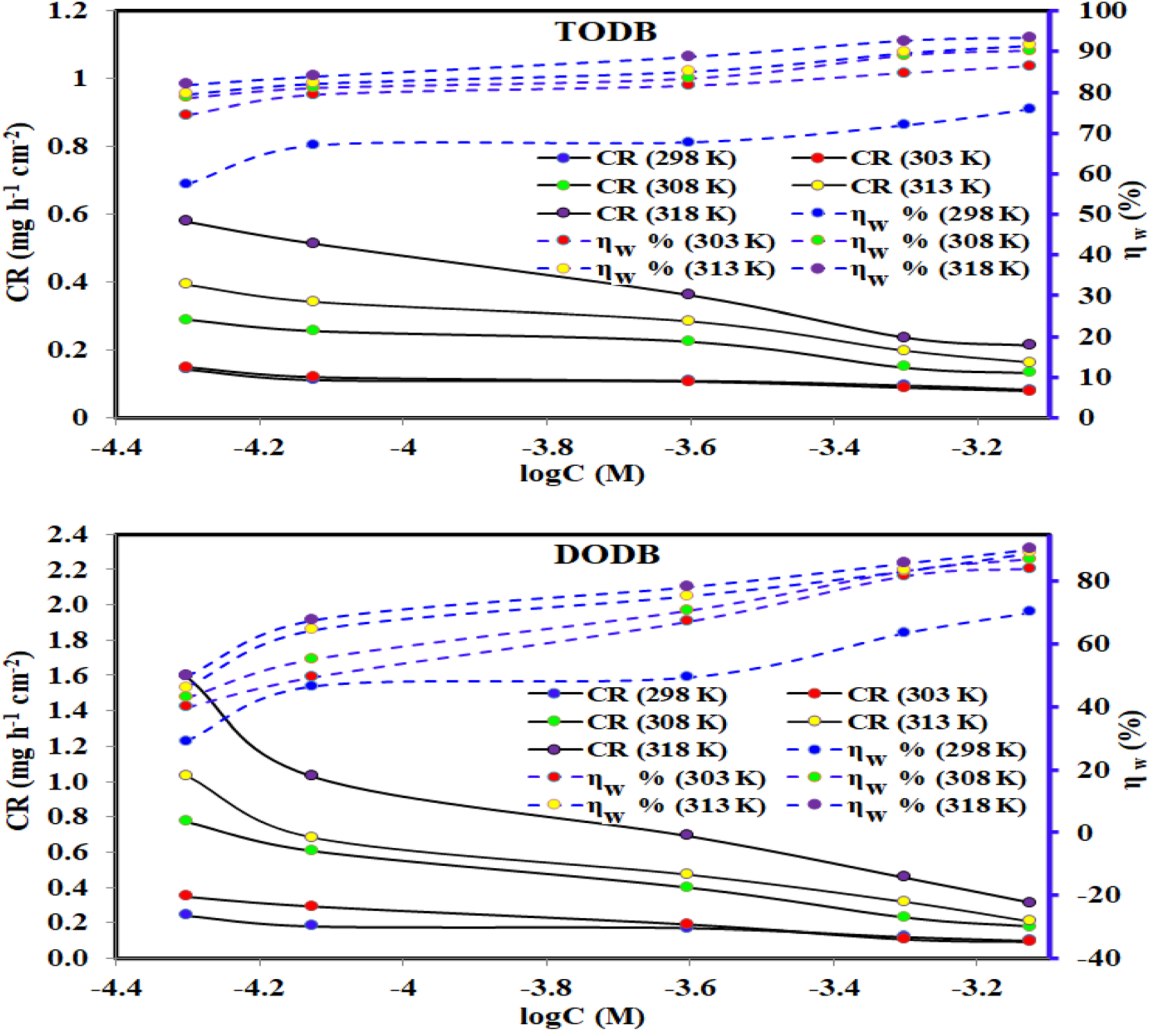
Effect of various temperatures and concentrations for synthesized inhibitors TODB and DODB on CR of steel in 1 M HCl using WL method.

The weight loss (WL) measurements of MS in HCl (1.0 M) solution were accomplished at different time intervals and temperatures (298 K–318 K) in the presence of corrosive medium-free inhibitors and 0.5 × 10^−4^ and 7.5 × 10^−4^ of TODB and DODB, respectively. Fig. 5 depicts the relationship among the arithmetic values for both the corrosion rate (*C*_R_) and the inhibition efficiency (percent IE) of the prepared TODB and DODB against log C at various temperatures (298 K–318 K). Table 2 also includes the average values of the *C*_R_, and percent IE of the studied organic compounds. Table 2 shows that as the inhibitor concentrations increased and the temperature remained constant, the values of *C*_R_ decreased while the percent IEs increased. This can be attributed to the increased adsorption coverage of the inhibitor molecules on the metal substrate with increasing concentrations, which decreased the MS dissolution rates. As an outcome, the investigated organic molecules are recognized as capable inhibitors of MS corrosion in HCl (1.0 M) solutions. In contrast, at constant inhibitor concentration and rising temperature, the value of *C*_R_ rose marginally while the percent IE grew exponentially. These research results unequivocally prove that the adsorption mechanism of prepared TODB and DODB on the metallic substrate in HCl (1.0 M) solutions was characteristic of chemical adsorption. Fiori-Bimbi *et al.*^36^ discovered similar results while inhibiting M − steel corrosion in HCl solutions with pectin. The values of % IE obtained for the inhibitor TODB were often greater than those for the inhibitor DODB. The physicochemical properties of the molecules, such as the functional groups, steric factor, molecular (size, weight, and structure) and aromaticity,^37–39^ determine the adsorption of any corrosion inhibitors. Since the investigated azo compounds (TODB and DODB) have these characteristics, they have a good adsorption capacity and thus can behave as effective corrosion inhibitors. By comparison with previous publications, it was noticed that the currently investigated compounds had higher percentage of IEs than other reported organic dyes as corrosion inhibitors of various steel alloys in acidic environment.^40–42^

**Table 2:** Corrosion rate (*C*_R_), surface coverage (*θ*) and percentage of inhibition efficiency (*η*) of MS in 1.0 M HCl of the TODB and DODB derivatives at different temperatures

Inhibitor	Inhibitor conc. (M)	25 °C			30 °C			35 °C			40 °C			45 °C		
		*C*_R_, (mg cm−2 h−1)	*θ*	*η*_w_ (%)	CR, (mg cm^−2^ h−1)	*θ*	*η*_w_ (%)	*C*_R_, (mg cm^−2^ h^−1^)	*θ*	*η*_w_ (%)	*C*_R_, (mg cm^−2^ h^−1^)	*θ*	*η*_w_ (%)	*C*_R_, (mg cm^−2^ h^−1^)	*θ*	*η*_w_ (%)
Blank	0.00 × 10^−4^	0.3365	—	—	0.5765	—	—	1.3534	—	—	1.9141	—	—	3.1736	—	—
TODB	0.50 × 10^−4^	0.1432	0.574	57.45	0.1479	0.743	74.34	0.2894	0.786	78.62	0.3917	0.795	79.54	0.5776	0.818	81.80
	0.75 × 10^−4^	0.1113	0.669	66.93	0.1194	0.793	79.29	0.2565	0.810	81.04	0.3418	0.821	82.14	0.5134	0.838	83.82
	2.50 × 10^−4^	0.1087	0.677	67.71	0.1065	0.815	81.53	0.2254	0.833	83.34	0.2846	0.851	85.13	0.3612	0.886	88.62
	5.00 × 10^−4^	0.0945	0.719	71.93	0.0882	0.847	84.69	0.1494	0.890	88.96	0.1976	0.897	89.68	0.2365	0.925	92.55
	7.50 × 10^−4^	0.0812	0.759	75.88	0.0783	0.864	86.42	0.1334	0.901	90.14	0.1622	0.915	91.52	0.2126	0.933	93.30
DODB	0.50 × 10^−4^	0.2389	0.290	29.00	0.3473	0.398	39.76	0.7732	0.429	42.87	1.0309	0.461	46.14	1.5949	0.497	49.75
	0.75 × 10^−4^	0.1802	0.464	46.44	0.2920	0.493	49.34	0.6071	0.551	55.14	0.6816	0.644	64.39	1.0283	0.676	67.60
	2.50 × 10^−4^	0.1707	0.493	49.28	0.1898	0.671	67.08	0.3978	0.706	70.61	0.4721	0.753	75.34	0.6908	0.782	78.23
	5.00 × 10^−4^	0.1230	0.635	63.46	0.1063	0.816	81.56	0.2277	0.832	83.17	0.3187	0.834	83.35	0.4545	0.857	85.68
	7.50 × 10^−4^	0.1006	0.701	70.10	0.0922	0.840	84.00	0.1759	0.870	87.00	0.2067	0.892	89.20	0.3124	0.902	90.16

#### 3.2.2. PDP measurements

**Fig. 6:**
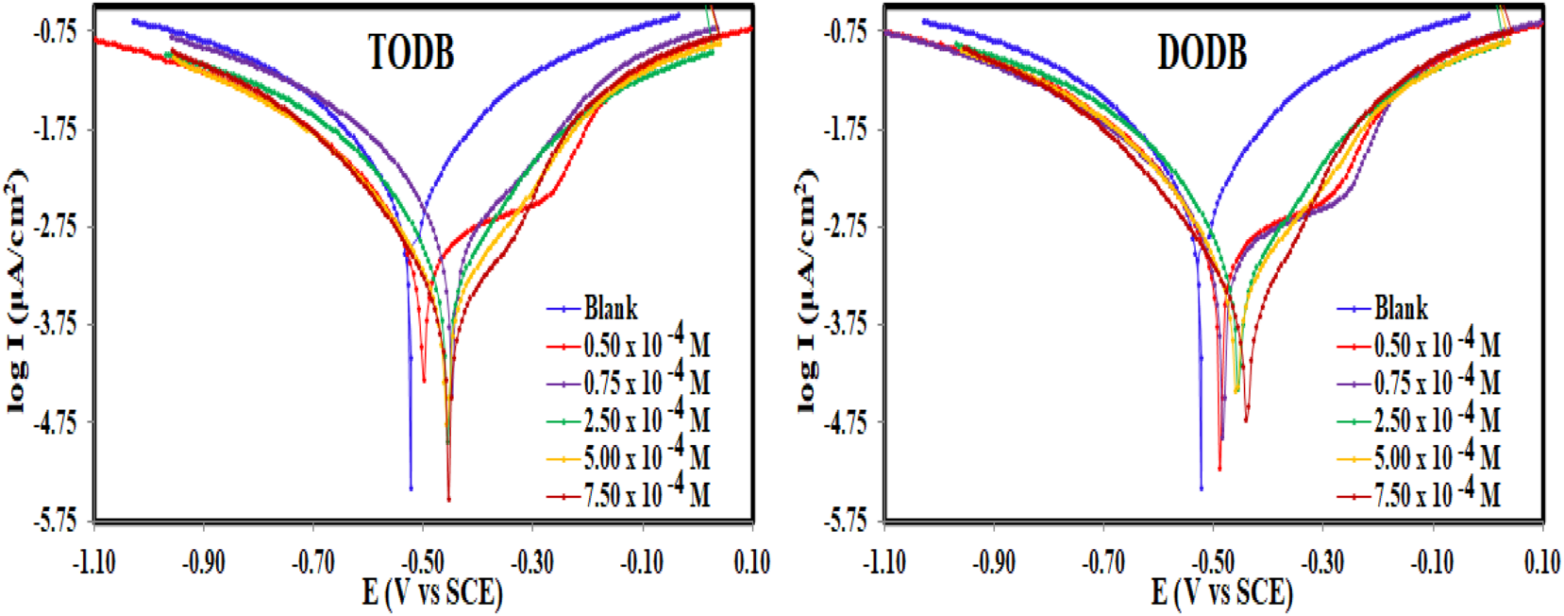
PDP curves for the corrosion of MS in 1 M HCl with and without different concentrations of synthesized inhibitors TODB and DODB at 25 °C.

This method was used to study both cathodic and anodic reactions of TODB and DODB at 298 K in HCl (1.0 M) without and with inhibitor on the MS electrode after performing the OCP test, and approaching the steady state potential. Fig. 6 shows the polarization profiles that were acquired. It was also found in the literature^43^ that when the corrosion potential (*E*_corr_) shift is greater than ±85 mV *vs.* the *E*_corr_ of the uninhibited sample, the inhibitor is classified as cathodic or anodic, while it is classified as mixed-type when the shift is less than ±85 mV. The introduction of TODB and DODB in HCl (1.0 M) solution markedly decreased the Tafel sections in the current study, and the movement in the *E*_corr_ was less than ±85 mV, indicating that the prepared azo derivatives are mixed-type inhibitors.^44^ The electrochemical variables like *E*_corr_, Tafel constants for both anode (*β*_a_) and cathode (*β*_c_), corrosion current density (*I*_corr_), polarization resistance (*R*_p_), (*θ*), (*C*_R_), and (% IE) were calculated from the Tafel graphs and are listed in Table 3.

**Table 3:** Electrochemical parameters^a^ for MS dissolution in 1.0 M HCl solution containing different concentrations of the (DODB–TODB) inhibitors obtained from PDP measurements at 30 °C

Inhibitor name	Conc. (M)	*E*_corr_ *vs.* SCE (mV)	*I*_corr_ (μA cm^−2^)	*β*_a_ (mV dec^−1^)	*β*_c_ (mV dec^−1^)	*C*_R_ (mpy)	*θ*	*η*_p_%
Blank	—	−523	8624	305	333	4137	—	—
DODB	0.50 × 10^−4^	−489	3990	607.1	599.7	1825	0.537	53.71
	0.75 × 10^−4^	−484	3290	578.9	564.1	1504	0.618	61.83
	2.50 × 10^−4^	−454	1540	230	243	702.9	0.821	82.13
	5.00 × 10^−4^	−458	945	211.3	217.6	431.6	0.890	89.04
	7.50 × 10^−4^	−439	553	175.6	204.7	252.8	0.936	93.58
TODB	0.50 × 10^−4^	−499	3730	625.2	590.2	1704	0.567	56.73
	0.75 × 10^−4^	−448	2560	239.6	259.2	1170	0.703	70.30
	2.50 × 10^−4^	−456	1330	232.7	245.9	606.7	0.846	84.57
	5.00 × 10^−4^	−457	713	201.7	217.6	325.9	0.917	91.73
	7.50 × 10^−4^	−454	436	177.6	192.5	199.1	0.949	94.94

a *E*_corr_ is the corrosion potential; *I*_corr_ is the corrosion current density: *β*_a_ and *β*_c_ are Tafel constants for both anode and cathode; (*C*_R_) is the corrosion rate; *θ* is the surface coverage; *η*_p_ is the inhibition efficiency.

By extrapolating the linear parts of the Tafel branches to the relevant values of (*E*_corr_), the values of (*I*_corr_) were computed. The analysis of the results showed that, in comparison to the uninhibited solution, the (*I*_corr_) and (*C*_R_) decreased in the presence of TODB and DODB, demonstrating that these substances were adsorbed on the MS surface and delayed the dissolving of the metal in the acidic environment (Table 3). Furthermore, when compared to the inhibitor-free sample, the azo derivatives reduced the oxidation reaction of MS and delayed the hydrogen ion reduction on the surface of the cathode. Moreover, the equivalent form of the Tafel curves suggests that the inhibition mechanism is activation-controlled in the existence of the evaluated inhibitors.^45^ The (*I*_corr_) values are much lower in the presence of the inhibitors (0.436 mA cm^−2^ for TODB and 0.55 mA cm^−2^ for DODB) than in the absence of the inhibitors (0.86 mA cm^−2^), with maximum efficiency (94.9% and 93.6%), respectively, at concentrations of 7.50 × 10^−4^ M for TODB and DODB, indicating that these materials mitigate corrosion and preserve MS. The involvement of more hetero atoms (S and N) with lone pair of electrons in the framework of the (TODB) molecule, which increases adsorption, may explain the higher *η* for the (TODB) molecule compared to the (DODB) molecule.

#### 3.2.3. EIS study

**Fig. 7:**
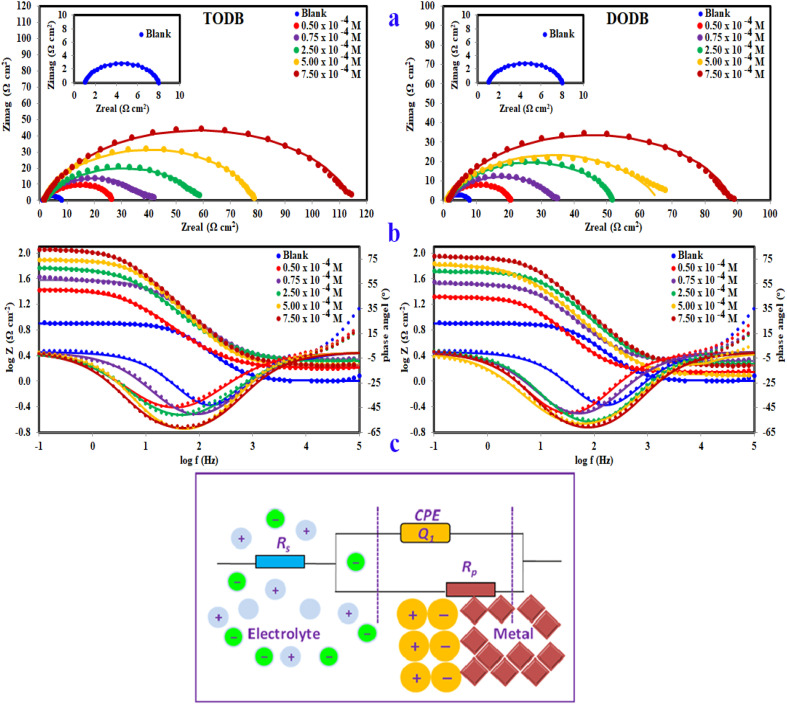
Nyquist plots (a), Bode & phase angle (b) and the equivalent circuit model (c) for fitting EIS data for MS in 1 M HCl with and without different concentrations of synthesized inhibitors TODB and DODB at 25 °C.

EIS readings were used to prove the previously mentioned corrosion behavior, as well as to investigate the capacitive characteristics at the MS/solution interface. The Nyquist and Bode plots of the MS in HCl (1.0 M) without and in the existence of different molarities of (TODB and DODB) are seen in Fig. 7a, after the OCP test and approaching the steady state potential. The documented Nyquist plots resembled each other, indicating that the addition of (TODB and DODB) inhibited MS corrosion without influencing the mechanism.^46^ At low frequency, the Nyquist plots are best described by only single semicircles, suggesting that the corrosion process is under charge transfer control.^47^ The increasing values of (*n*) (Table 4) for (TODB and DODB) *versus* the blank sample clearly show that the inhibitor increases surface uniformity *via* adsorption. Fig. 7b shows the correlating Bode plots for the MS electrode immersed in HCl (1.0 M) with and without various concentration levels. As shown by these figures, there is an increase in the absolute impedance |Z| at low frequencies. This increase confirms the higher (percent *η*) obtained at high concentration, which is due to adsorption of (TODB and DODB) on the MS surface and blocking its active sites. Furthermore, the shift in phase angle values in the negative direction demonstrates that TODB and DODB primarily perform by providing a thin film over the MS surface.^48,49^

**Table 4:** EIS parameters for corrosion of MS in 1.0 M HCl in the absence and presence of different concentrations of (DODB–TODB) inhibitors at 30 °C^a^

Inhibitor	Conc. (M)	*R*_s_ (*R*_u_) (Ω cm^2^)		*R*_ct_ (*R*_p_) (Ω cm^2^)		*Y*_o_ (μΩ ^−1^ s^n^ cm^−2^)			*n*	*C*_dl_ (μF cm^−2^)		Chi squared	*S*	*α*	*θ*	*η*_z_ %
Blank	—		1.082		6.884		478.50	0.8836			225.363	2.25 × 10^−2^	−0.365	−42.07	—	—
DODB	0.50 × 10^−4^		1.385		19.42		2129.00	0.8493			1209.689	9.14 × 10 ^−3^	−0.449	−49.67	0.6455	64.55
	0.75 × 10^−4^		2.077		31.98		1049.00	0.8299			523.103	4.97 × 10 ^−3^	−0.511	−49.78	0.7847	78.47
	2.50 × 10^−4^		1.877		50.2		536.60	0.8453			276.941	3.99 × 10 ^−3^	−0.627	−55.48	0.8629	86.29
	5.00 × 10^−4^		1.203		64.43		128.10	0.7983			38.121	1.55 × 10^−3^	−0.652	−58.38	0.8932	89.32
	7.50 × 10^−4^		1.761		86.56		457.50	0.8416			249.150	4.74 × 10 ^−3^	−0.679	−60.15	0.9205	92.05
TODB	0.50 × 10^−4^		1.601		26.16		2717.00	0.757			1162.759	6.88 × 10 ^−3^	−0.426	−44.38	0.7369	73.69
	0.75 × 10^−4^		2.149		36.49		635.30	0.8237			283.835	6.16 × 10 ^−3^	−0.564	−50.62	0.8113	81.13
	2.50 × 10^−4^		2.066		57.41		1164.00	0.77			518.754	5.30 × 10 ^−3^	−0.523	−51.41	0.8801	88.01
	5.00 × 10^−4^		1.807		77.04		548.40	0.8708			342.934	4.82 × 10 ^−3^	−0.662	−61.68	0.9106	91.06
	7.50 × 10^−4^		1.773		112.9		598.40	0.8328			348.361	5.03 × 10 ^−3^	−0.662	−61.31	0.9390	93.90

a *R*_s_ = solution resistance, *R*_ct_ = charge transfer resistance, *Y*_0_, *n* = constant phase elements, *C*_dl_ = double layer capacitance, *θ* = surface coverage, *η*_*z*_ = inhibition efficiency.

Fig. 7a and b show a comparison of the experimental EIS data recorded for (TODB and DODB) in the presence of various concentrations in comparison to the blank. The well-fitted data were collected by employing the equivalent circuit shown inset (Fig. 7c), where *R*_s_ (the solution resistance), *R*_ct_, and *C*_dl_ are recorded in Table 4. The EIS variables determined with this circuit indicate that *R*_ct_ is directly related to the molarity of the inhibitor, whereas the (*C*_dl_) values show the inverse correlation. This concept could be caused by either H_2_O molecule desorption from the surface of MS or inhibitor adsorption on the metal substrate.^50,51^ In agreement with previous techniques, the % *η* of the evaluated azo derivatives is as follows: (TODB) > (DODB), with optimum inhibition values of 93.9% and 92.05%. This EIS-observed attitude corresponds to the polarization data. The electron donating impact of the sulfonyl (–SO_3_H) and hydroxyl (–OH) groups bonded to the aromatic ring increases the electron density on the benzene ring, inhibiting the corrosion process.

#### 3.2.4. EFM technique

EFM is a nondestructive technique that can provide (*I*_corr_) values without the need for previous information of Tafel constants (*β*_c_ and *β*_a_). EFM, like EIS, is a technique that employs small AC signals. In contrast to EIS, two sine waves (at different frequencies) are implemented to the cell all at once. *I*_corr_ can be calculated from the intermodulation spectra at the tallest altitude employing EFM instead of using Tafel slope values (*β*_c_ and *β*_a_). We can also use the causality factors [CF-2 and CF-3] to verify the information generated by this technique.^52^ The EFM intermodulation spectra are a relationship of the current feedback as a function of input frequency that includes current feedback reflecting harmonic and intermodulation peaks. All kinetic components derived from EFM, such as corrosion current, Tafel slopes, and causality factors, were calculated using the highest peaks and are shown in Table 4. EFM measurements were used to calculate the (% *η*) and (*θ*) of the studied compounds from the following equation:^53^

14 % *η*_EFM_ = *θ* × 100 = [(*i*^o^_corr_ − *i*co_rr_)/*i*^o^_corr t_] × 100

**Fig. 8:**
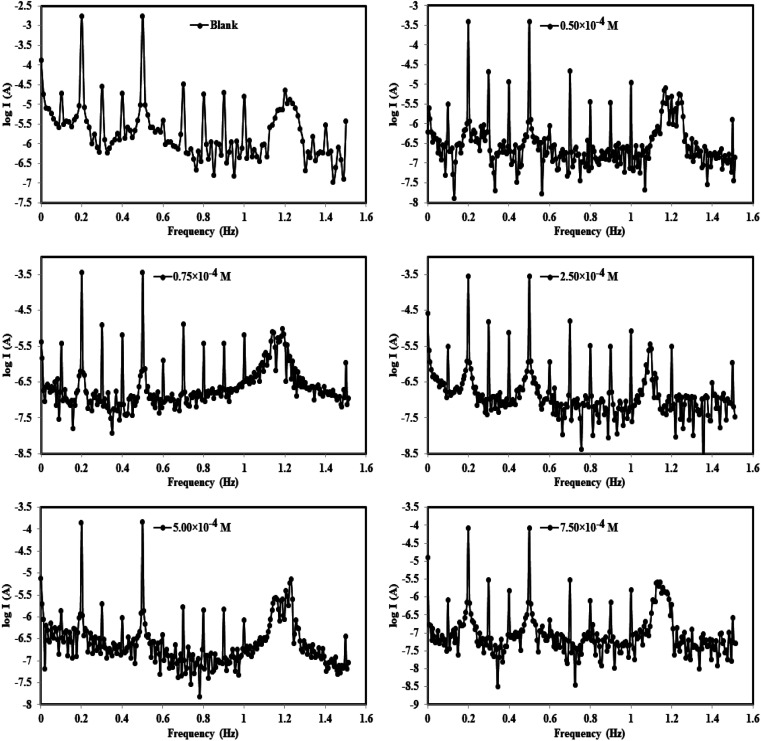
EFM curves for the corrosion of MS in 1 M HCl with and without different concentrations of synthesized inhibitor (DODB for example) at 25 °C.

The intermodulation spectra of the two prepared azo derivative (TODB and DODB) obtained in this study are shown in Fig. 8. The data in Table 5 show that the *i*_corr_ values for the studied molecules are lower than those for the aggressive solution HCl (1.0 M), indicating that they inhibited corrosion. It was also discovered that *i*_corr_ changed with concentration, reaching its lowest value at the highest concentration (7.50 × 10^−4^ M), indicating that the (*η*_EFM_ percent) increases with azo derivative concentrations. Table 5 also shows that the surface coverage values of the TODB molecules are greater than those of DODB, implying that the inhibition of TODB is greater than that of DODB. Finally, as a result, we can conclude and guarantee the method's and result's validity.

**Table 5:** Electrochemical kinetic parameters^a^ obtained by EFM technique for MS in the absence and presence of various concentrations of (DODB–TODB) inhibitors in 1.0 M HCl at 30 °C

Inhibitor name	Conc. (M)	*I*_corr_ (μA cm^−2^)		*β*_a_ (mV dec^−1^)	−*β*_c_ (mV dec^−1^)	CF-2	CF-3	*k* (mpy)	θ	*η*_EFM_%
Blank	—	2791	100.4		113.1	1.763	3.155	1275.00	—	—
DODB	0.50 × 10^−4^	755.2	103.4		158.5	1.905	3.534	345.1	0.7294	72.94
	0.75 × 10^−4^	649.8	103.5		133.1	1.976	3.136	296.90	0.7672	76.72
	2.50 × 10^−4^	496.8	96.22		142.8	1.956	2.789	227.00	0.8220	82.20
	5.00 × 10^−4^	270.1	116.5		128.1	2.009	3.467	123.4	0.9032	90.32
	7.50 × 10^−4^	166.3	112.2		148.3	1.958	2.925	76.01	0.9404	94.04
TODB	0.50 × 10^−4^	694.4	108.2		131.1	1.948	2.930	317.30	0.7512	75.12
	0.75 × 10^−4^	550.4	119.5		192.6	1.967	3.061	251.50	0.8028	80.28
	2.50 × 10^−4^	416.1	100		109.9	2.143	3.093	190.1	0.8509	85.09
	5.00 × 10^−4^	203.4	100.8		105.5	1.547	3.76	92.94	0.9271	92.71
	7.50 × 10^−4^	133.3	114.3		146.9	1.980	2.914	60.91	0.9522	95.22

a E_corr_ is the corrosion potential; *I*_corr_ is the corrosion current density: *β*_a_ and *β*_c_ are Tafel constants for both anode and cathode; *k* is the corrosion rate; *θ* is the surface coverage; *η*_EFM_ is the inhibition efficiency.

### 3.3. Adsorption isotherm

**Fig. 9:**
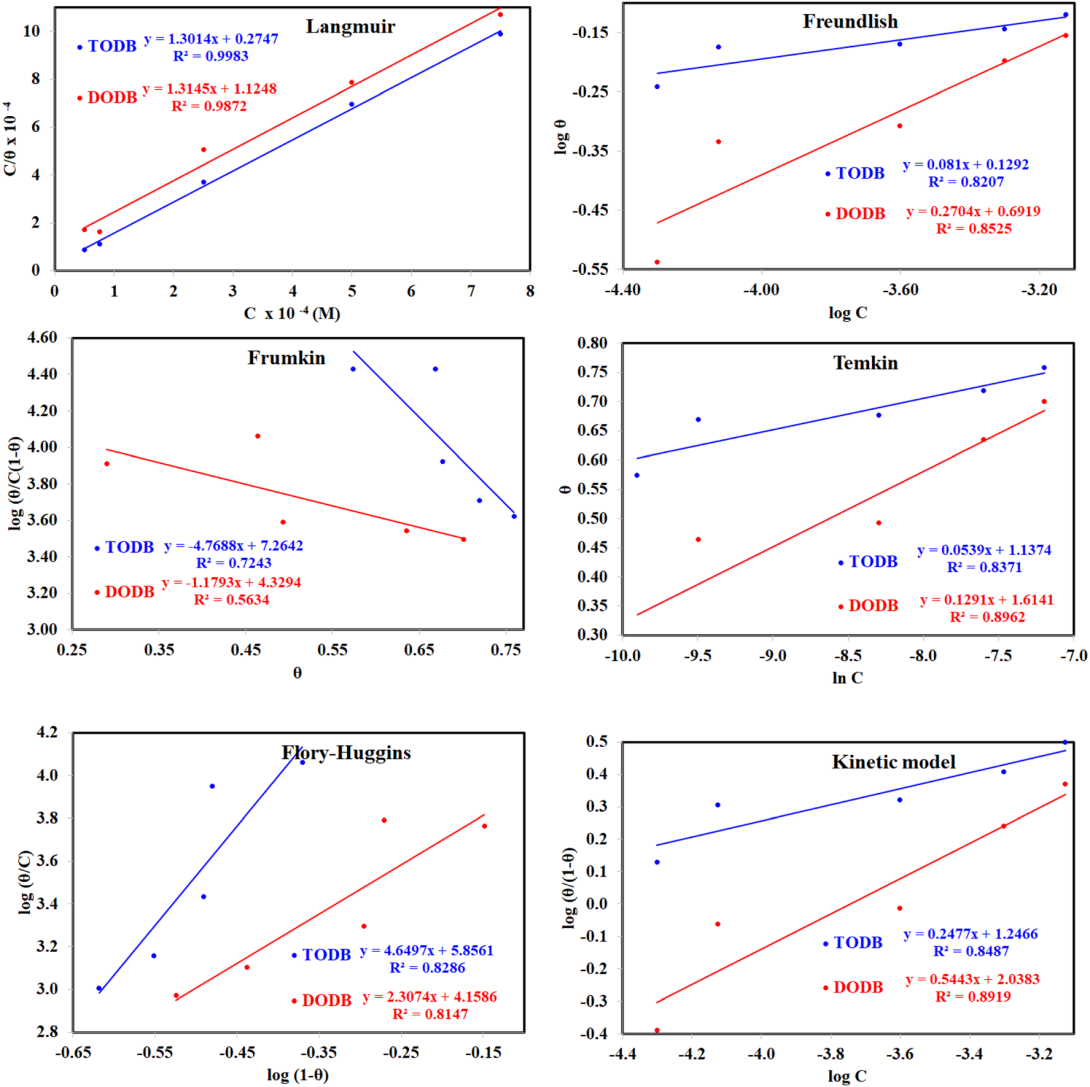
Different adsorption isotherms for synthesized inhibitors TODB and DODB using WL method.

The examined TODB and DODB involve heteroatoms like O, S, and N in TODB and O and S in DODB, as well as aromatic rings and azo groups that can be adsorbed on the steel surface to generate protective layers.^53^ These layers can be created using one of the adsorption modes listed as follows:^54^ (1) physical: arises as a result of electrostatic forces between the protonated groups of organic molecules and the charged metal surface; (2) chemical: by forming coordination bonds between the vacant d orbital of the substrate and the lone pair of electrons of the heteroatoms; or (3) integration of two adsorption types. To observe and analyze the better adsorption isotherm (Langmuir, Flory–Huggins, Temkin, Frumkin, Freundlich type and Kinetic model) of experimented azo compounds, graphs of each adsorption isotherms were evaluated separately (Fig. 9). The regression correlation coefficient (*R*^2^) values listed in Table 6 show that the adsorption behavior of TODB and DODB on the MS surface correlated well with the Langmuir isotherm,^55^ which is given by the formula:^56^

15 *C*_inh_/*θ* = (1/*K*_ads_) + *C*_inh_

where *C*_inh_ is the molarity of the tested TODB and DODB inhibitors, *K*_ads_ is the adsorption–desorption equilibrium constant for the metal substrate processes, and (*θ*) is estimated using electrochemical methods. The graph of *C* against *C*/*θ* demonstrates a straight fitting line with a slope and correlation coefficient near 1 (Fig. 9). Formula (8) was used to quantify (*K*_ads_) from the standard free energy of adsorption (
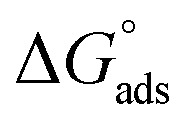
):^57^16
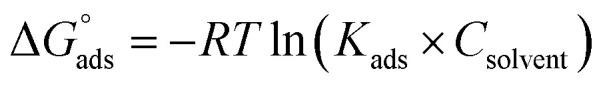
where *C*_solvent_ denotes the molarity of water in solution, *T* denotes the absolute temperature, and *R* denotes the universal gas constant. Table 6 shows the thermodynamics for the adsorption process of the experimented molecules (TODB and DODB). The fact that the 
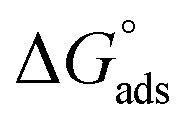
 is negative implies that the tested molecules (TODB and DODB) adsorb spontaneously on the surface of MS. Furthermore, higher *K*_ads_ values indicate a strong adsorption property, and as a result, a better inhibition property.^58^ In general, absolute values of 
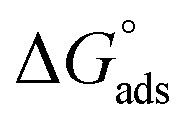
 of (−20 kJ mol^−1^) or less indicate electrostatic interaction (*i.e.*, physisorption). Values around −40 kJ mol^−1^ or more negative values, on the other hand, indicate charge sharing and bond formation (chemisorption),^59^ although there is little difference between the 
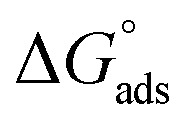
 values (between −20 and −40 kJ mol^−1^). This shows that TODB and DODB are physically and chemically adsorbed on the surface of MS, with physical adsorption having a particularly strong adsorption effect.^60^ It also appeared from Table 6 that the *K*_ads_ values of TODB molecules are higher than that of DODB, which can be interpreted as the inhibition of TODB < DODB.

**Table 6:** Adsorption isotherm models of the inhibitors^a^ with values of *R*^2^, slopes, intercepts, *K*_ads_, and 
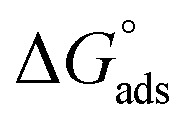
 obtained by using data from WL measurements

Adsorption isotherm model	Linear form equation	Temp °C	Inhibitor	Slope	Intercept	*R* ^2^	*K*_ads_ M^−1^	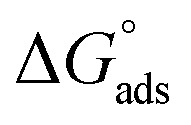 kJ mol^−1^
Freundlich	log *θ =* log *K +* 1/*n* log *C*	25	TODB	0.08096	0.12918	0.82068	1.3464	−10.87
			DODB	0.27036	0.69190	0.85250	4.9193	−14.13
Langmuir	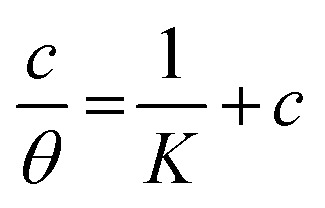	25	TODB	1.30135	0.00003	0.99834	36 404	−36.57
			DODB	1.31450	0.00011	0.98720	8891	−33.02
		30	TODB	1.14668	0.00001	0.99975	78 575	−38.51
			DODB	1.08781	0.00008	0.99834	12 878	−33.96
		35	TODB	1.09560	0.00001	0.99953	71 240	−38.27
			DODB	1.06735	0.00007	0.99858	14 909	−34.33
		40	TODB	1.08096	0.00001	0.99960	72 951	−38.33
			DODB	1.06596	0.00005	0.99814	18 864	−34.92
		45	TODB	1.05884	0.00001	0.99987	88 377	−38.81
			DODB	1.05944	0.00004	0.99893	22 265	−35.34
Frumkin	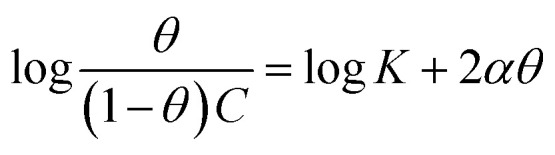	25	TODB	−4.76876	7.26419	0.72434	1.8374 × 10^7^	−52.25
			DODB	−1.17930	4.32940	0.56337	2.1350 × 10^4^	−35.23
Temkin	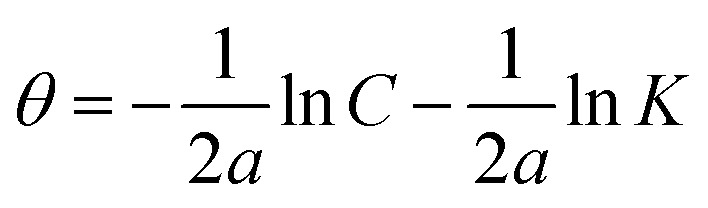	25	TODB	15.54114	−19.06014	0.83714	0.2933	−7.03
			DODB	6.93990	−12.08353	0.89625	0.1753	−5.73
Flory–Huggins	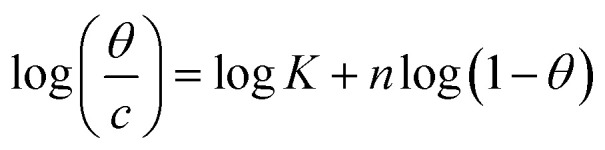	25	TODB	4.64966	5.85609	0.82861	7.1794 × 10^5^	−44.09
			DODB	2.30738	4.15856	0.81466	1.4407 × 10^4^	−34.24
Kinetic-thermodynamic	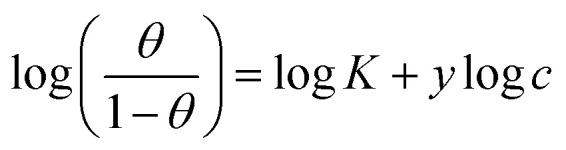	25	TODB	0.24766	1.24663	0.84871	17.6452	−17.35
			DODB	0.54430	2.03829	0.89192	109.2168	−21.94

a *R* ^2^ = regression correlation coefficient, *K*_ads_ = binding constant, *θ* = surface coverage, *C* = concentration.

### 3.4. Effect of temperature and activation parameters

The activation energy (*E*_a_) and other thermodynamic activation functions can be calculated using mass loss experiments at different temperatures with and without inhibitors. The findings can help to explain the mechanism of inhibition. This study adds to our understanding of the adsorption mechanism by analyzing the thermodynamic parameters for MS dissolution in HCl (1.0 M) without and in the presence of different molarities of TODB and DODB. The Arrhenius and transition-state equations^61^ were used to obtain these thermodynamic functions:

17 *C*_R_ = *A* exp(−*E*_a_/*RT*)

**Fig. 10:**
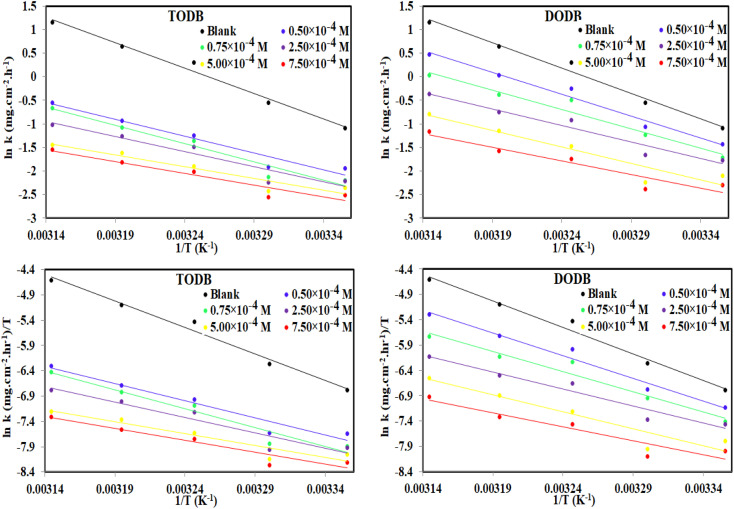
Arrhenius and Transition state plots for MS dissolution with and without various dosages of synthesized inhibitors TODB and DODB in 1 M HCl solution.

18
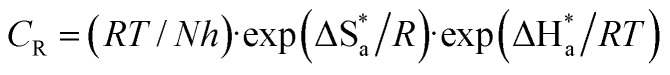
where 
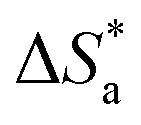
 and 
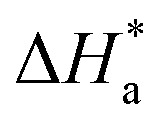
 are the apparent entropy of activation and enthalpy of activation, respectively, and *h* and *N* are Planck's constant and Avogadro's number, respectively. In the case of heterogeneous chemical reactions, the constant A is known as the pre-exponential factor because it is related to the number of active centers. For MS in HCl (1.0 M) solutions containing different molarities of the two investigated inhibitors in the temperature range of 298 K to 318 K, the Arrhenius and transition state plots were constructed using chemical and electrochemical measurements. The Arrhenius and transition state plots of MS in HCl (1.0 M) solution without and with different molarities (0.50 × 10^−4^ to 7.50 × 10^−4^ M) are shown in Fig. 10. Table 7 shows the values of the thermodynamic activation functions calculated from these plots as a function of the inhibitor concentration. The rise in inhibition efficiency values with increasing temperature (see Table 2), and the gradual decrease in 
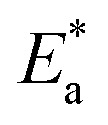
 with increasing molar concentration of the inhibitor (Table 7) can be explained on the basis of chemical adsorption. According to Amin *et al.*,^60^ it is clear from the data in Table 6 that the process of MS dissolution is defined by 
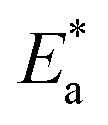
, which is lower in the presence of these molecules (TODB and DODB) than it is in the uncontrolled HCl (1.0 M). A review of the results in Table 10 showed that the activation parameters 
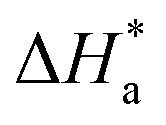
 and 
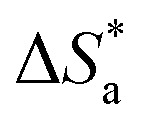
 of the MS dissolving reaction are higher in their absence than in their presence. The positive sign for the enthalpy value indicates that the process of dissolving steel is endothermic, which makes it challenging.^62^ As shown in Table 11, values for (
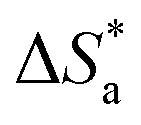
) increased in both tested media when the TODB and DODB were present relative to a free aggressive solution. Such fluctuation is connected to the phenomenon of inhibitor molecule ordering and disordering on the MS surface. According to the growing 
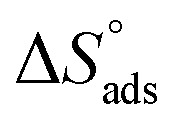
 in the presence of the inhibitor, disordering is intensified as the reactant moves from the reactant to the activated complex. In other words, the catalyst for the adsorption of the inhibitor onto the MS surface is the rise in entropy that occurs throughout the adsorption process.^63^

**Table 7:** Activation parameter values for steel in 1.0 M HCl in the absence and presence of different concentrations of the TODB and DODB derivatives

Inhibitor	Conc., (M)	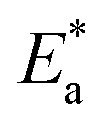 kJ mol^−1^	ΔH* kJ mol^−1^	ΔS* J mol^−1^ K^−1^
Blank	0.00 × 10^−4^	89.76	87.20	38.86
TODB	0.50 × 10^−4^	59.20	56.64	−72.14
	0.75 × 10^−4^	64.69	62.13	−55.70
	2.50 × 10^−4^	53.32	50.76	−93.96
	5.00 × 10^−4^	41.54	38.99	−134.75
	7.50 × 10^−4^	41.73	39.17	−135.30
DODB	0.50 × 10^−4^	77.06	74.50	−6.91
	0.75 × 10^−4^	68.42	65.86	−37.54
	2.50 × 10^−4^	58.40	55.85	−72.86
	5.00 × 10^−4^	58.33	55.77	−76.90
	7.50 × 10^−4^	48.29	45.73	−111.86

### 3.5. ICPE test

The corrosion rate for the two synthetic inhibitors TODB and DODB was determined by quantifying the total iron ions for the MS after immersion for 24 hours in HCl corrosive medium (1.0 M) at various doses of prepared inhibitors (0.5, 0.75, 2.5, 5, and 7.5 × 10^−4^ M), as well as for the MS in the exact corrosive medium with no inhibitors. The total concentration of iron ions in solution for MS in HCl (1.0 M) without any inhibitor is 3104 ppm, and 468 ppm and 546 ppm for TODB and DODB at higher tested concentrations, respectively. By increasing the concentration of TODB and DODB, as demonstrated in Table 8, the effectiveness of inhibition increased.

**Table 8:** Determination of iron concentration (ppm) using ICPE in 1.0 M HCl without and with different concentrations of TODB and DODB derivatives at room temperature

Inhibitor	[Inhibitor] M	Total iron ions in solution (ppm)	*C*_R_ mg cm^−2^ hr^−1^	*θ*	*η*_ICPE_%
Blank	—	3104	3.685	—	—
TODB	0.50 × 10^−4^	792	0.940	0.7448	74.48
	0.75 × 10^−4^	640	0.760	0.7938	79.38
	2.50 × 10^−4^	600	0.712	0.8067	80.67
	5.00 × 10^−4^	548	0.651	0.8235	82.35
	7.50 × 10^−4^	468	0.556	0.8492	84.92
DODB	0.50 × 10^−4^	1540	1.828	0.5039	50.39
	0.75 × 10^−4^	1442	1.712	0.5354	53.54
	2.50 × 10^−4^	840	0.997	0.7294	72.94
	5.00 × 10^−4^	670	0.795	0.7841	78.41
	7.50 × 10^−4^	546	0.648	0.8241	82.41

### 3.6. UV-analysis

**Fig. 11:**
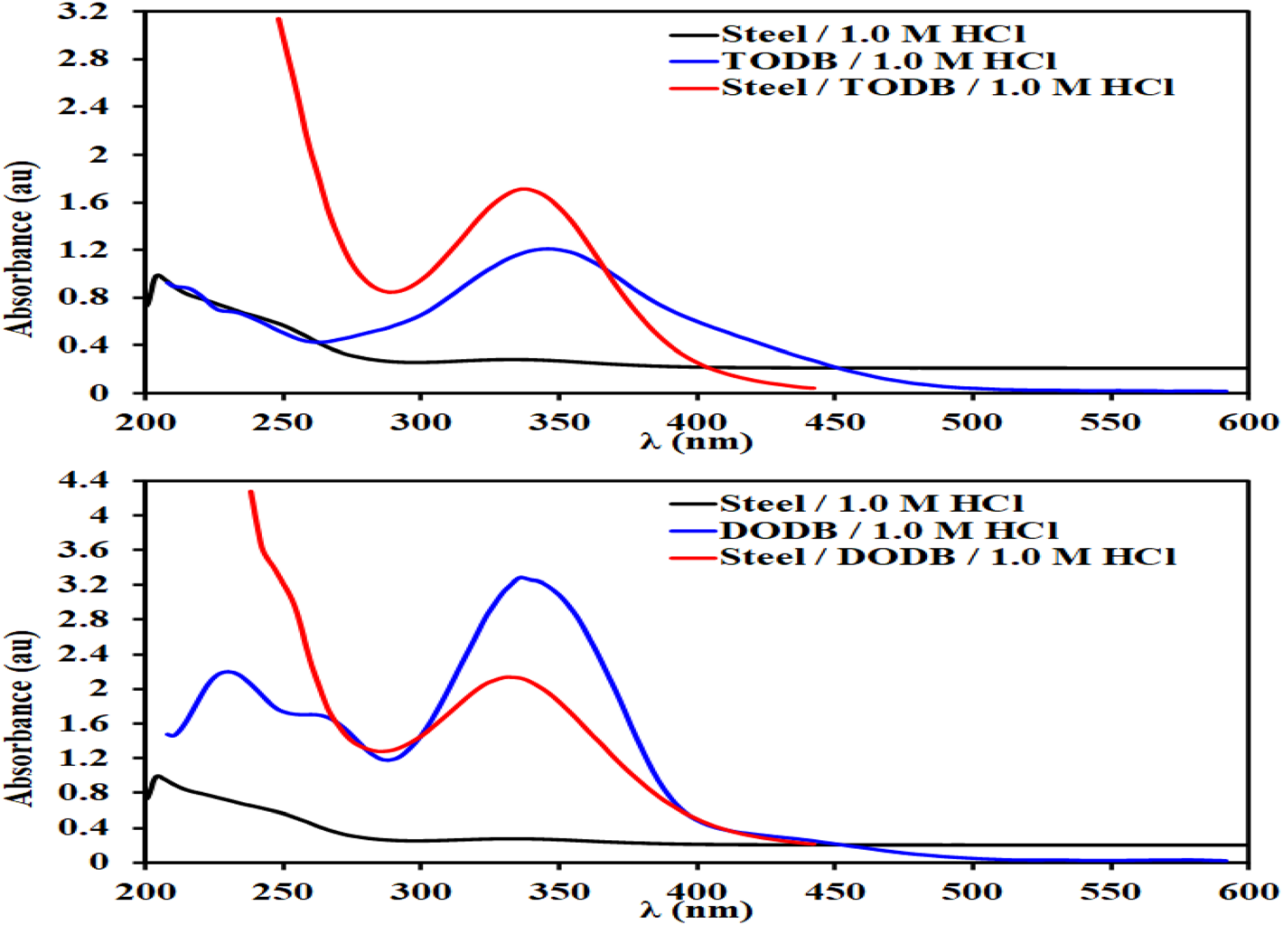
UV-visible spectra of MS in HCl, inhibitor + HCl and MS + inhibitor + HCl at 25 °C after immersion for 24 h.

Metal and inhibitor molecule interactions were studied *via* UV-visible spectroscopy^64^ for steel in HCl (1.0 M) without and in the presence of 7.50 × 10^−4^ M of the investigated inhibitors (TODB and DODB) at 25 °C after immersion for 24 h, in addition to 7.50 × 10^−4^ M of inhibitors in HCl (1.0 M) prior to immersion of MS. The UV-visible absorption spectra were recorded and the results are shown in Fig. 11. Following a 24 hour immersion in HCl, a typical band for steel at 205 nm (1 molar) was obtained. The levels of absorbance were adjusted to 340 nm and 336 nm for TODB and DODB, respectively, when 7.50 × 10^−4^ M of the two tested inhibitors were immersed with MS in HCl (1.0 M) for 24 hours. Due to the creation of a protective covering from the TODB and DODB molecules on the MS surface, the variation in the absorption values can provide conclusive proof for interaction between the evaluated inhibitors and the steel surface.

### 3.7. SEM and EDX spectroscopy

**Fig. 12:**
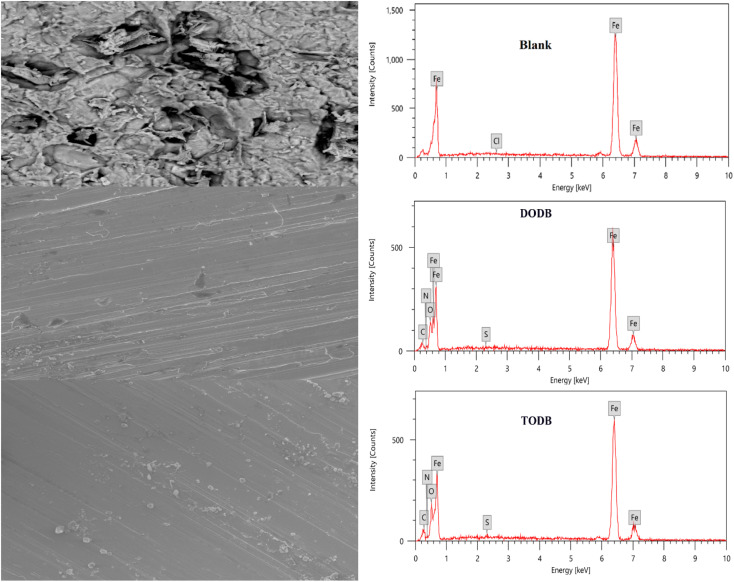
SEM images and EDX spectra for MS surface after immersion in 1.0 M HCl for 24 h in the absence and presence of synthesized inhibitors TODB and DODB at 25 °C.

To evaluate whether the surface morphology was altered by employing a certain concentration of the TODB and DODB molecules, the MS surface was investigated using SEM. The steel was examined following a 24 hour immersion in HCl (1.0 M) both with and without the use of 7.5 × 10^−4^ M from TODB and DODB. Without any inhibitor, corrosion severely degraded the surface of the MS in HCl (1.0 M). When the MS surface was examined after employing the inhibitors, it was smooth and pit-free (Fig. 12). We can infer from the SEM results that when the inhibitors were used, a protective coating formed on the MS surface. The inhibition efficiency studies conducted using chemical and electrochemical methods demonstrate the protective nature of this film. Additional proof of the components adhering to the surface of MS following immersion in corrosive solution with and without inhibitors can be determined using the EDX technique. However, when the steel surface was analyzed for samples immersed with 7.5 × 10^−4^ M of the TODB and DODB inhibitor molecules, respectively, new signals for O, S, N, and C were detected with significant concentration (Table 9) due to the formation of a protective layer on the surface after using the inhibitors (Fig. 12). In the case of steel immersed in HCl (1.0 M) alone, only Fe and Cl can be detected. These findings strongly support the use of TODB and DODB as MS corrosion inhibitors in HCl.

**Table 9:** Quantitative analysis for the MS surface after 24 hours immersion in 1.0 M HCl in the presence and absence of the prepared inhibitors (TODB and DODB) from EDX

Element	Blank (HCl–Fe)		DODB–Fe		TODB–Fe	
	Mass%	Atom%	Mass%	Mass%	Mass%	Atom%
Cl	0.06	0.10	—	—	—	—
C	—	—	4.09	13.49	6.45	20.06
N	—	—	1.16	3.27	0.46	1.23
O	—	—	9.06	22.44	9.68	22.62
S	—	—	0.07	0.09	0.55	0.64
Fe	99.94	99.90	85.61	60.71	82.86	55.45
Total	100	100	100	100	100	100

### 3.8. Biological activity

A sample solution containing Sulfate Reducing Bacteria (SRB) from one of the networks of fire hydrants on the Zohr Gas Field in Egypt was used for this test. The SRB BART vials were filled with 15 ml of this water, both with and without the addition of 1 ml of the tested inhibitors at a concentration of 1 ppm mol (TODB and DODB). For the entire test period of 11 days, the color to first black sign was monitored daily in order to estimate the matching population SRB reading shown in Table 10. The first black sign for the blank sample, which was not inhibited, appeared after 4 days of incubation with a population of roughly 27 000 SRB cfu ml^−1^, and it is regarded as aggressive. When the tested inhibitors were used, the first black indication for DODB and TODB showed after 7 and 8 days, respectively. According to Table 10, the approximate SRB populations were 325 cfu ml^−1^ and 75 cfu ml^−1^ for DODB (considered moderate) and TODB (considered low), respectively. These findings suggest that SRB can reduce corrosion formation, indicating that the inhibitor compounds TODB and DODB have good biological activity against SRB.^65^

**Table 10:** Approximate SRB Population for tested inhibitors

Inhibitor	Days of reaction	Approximate SRB Population (cfu ml^−1^)	
Blank	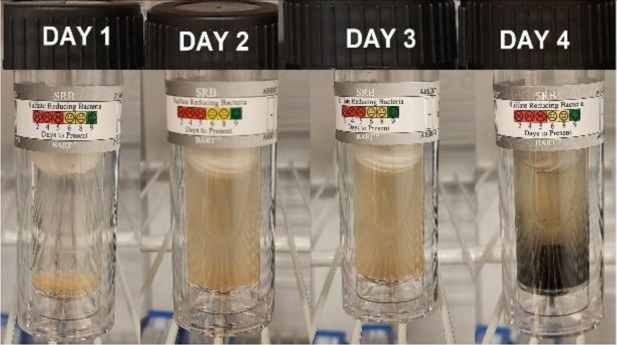	27 000	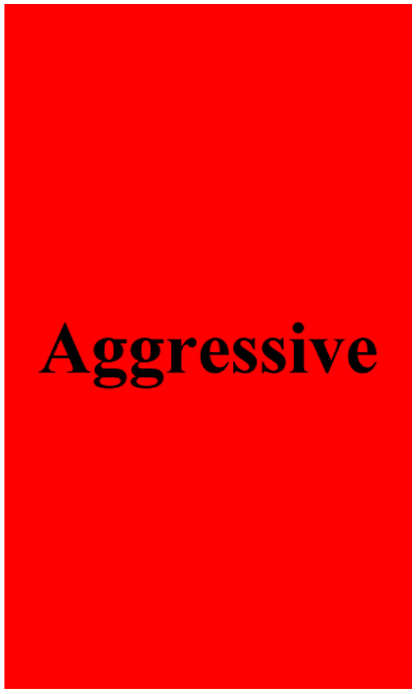
DODB	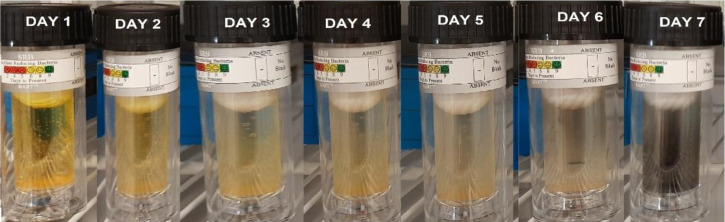	325	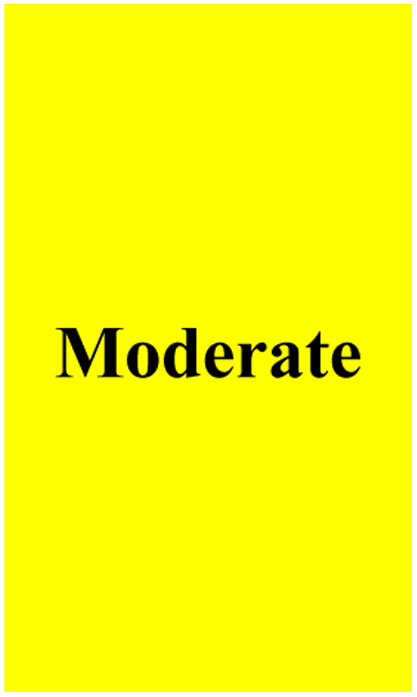
TODB	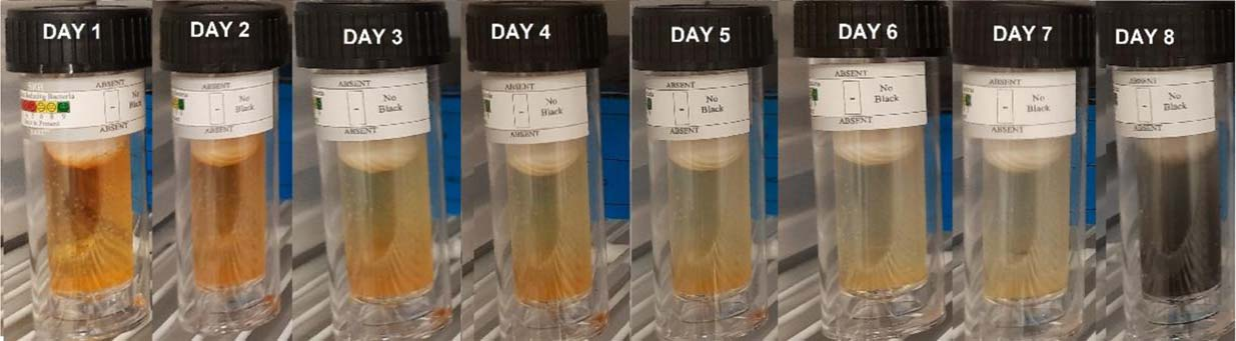	75	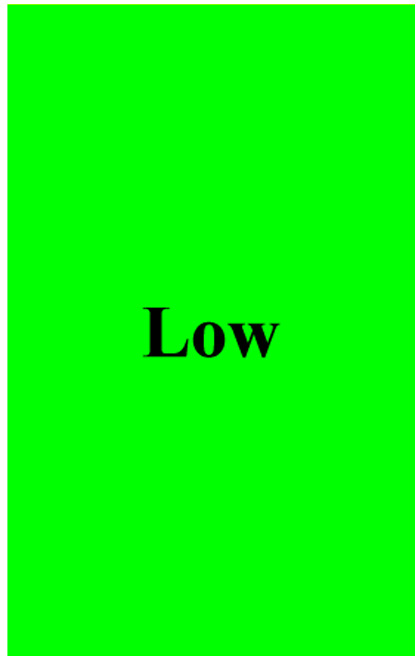

### 3.9. Relation between quantum calculations and corrosion inhibition

**Fig. 13:**
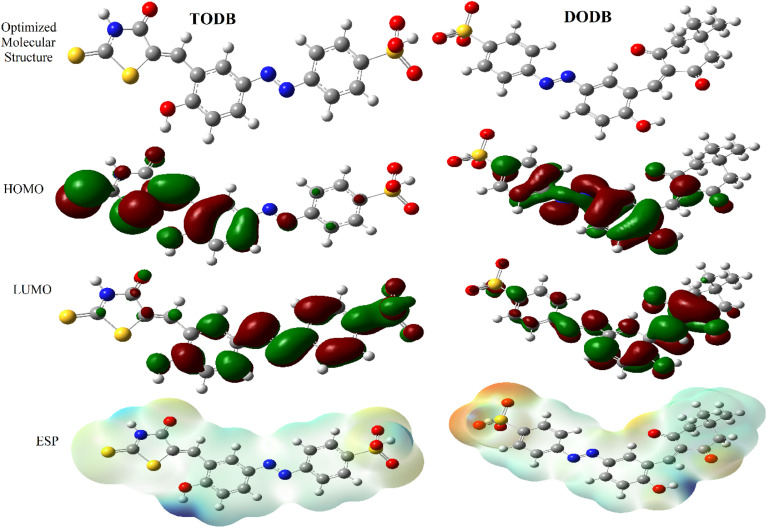
Optimized structures, HOMO, LUMO and ESP for synthesized inhibitors TODB and DODB using DFT/B3LYP/6-311 + G.

Various theoretical quantum chemical parameters were calculated using different methods to investigate the adsorption behavior and interaction between MS and the synthesized inhibitors TODB and DODB, semi-empirical PM6, HF-631G, DFT/B3LYP/6-311+G and MP2-6-311G basis sets were applied.^66,67^ The resulting values for the different calculated parameters are summarized in Table 11. Fig. 13 also shows the optimized molecular structures, HOMO, LUMO and ESP obtained from the DFT calculations with the B3LYP/6-311+G basis set. The HOMO is typically used to show how well an organic molecule can transfer electrons. In general, the *E*_HOMO_ value increases with the strength of a molecule's ability to donate electrons.^68^ From the summarized results in Table 11, the *E*_HOMO_ values equal −9.1237, −8.8766, −6.7038 and −9.1057 eV for TODB, and also equal −9.4758, −8.8374, −6.8554 and −8.8807 eV for DODB using the semi-empirical PM6, HF-631G, DFT/B3LYP/6-311+G and MP2-6-311G basis sets, respectively. From these results, it is clear that TODB has higher *E*_HOMO_ values than DODB *via* semi-empirical PM6 and DFT/B3LYP/6-311+G basis sets. As a result of this, it has the greatest ability to donate electrons to the MS surface, indicating that a protective film will be formed more readily with TODB than with DODB. It is known that the numerical number resulting from the difference between the LUMO and HOMO energies is called the energy gap, Δ*E*. As motioned in Table 11, TODB has the lowest Δ*E* values. As a result of this, it can deposit successfully on the mild steel surface more readily than DODB because the electrons located in the outer orbitals will be easier to donate.^69^ The Δ*E* values equal 7.2235, 9.0785, 3.1274 and 8.9139 eV for TODB, and 7.9966, 9.3270, 3.4741 and 9.0524 eV for DODB using the semi-empirical PM6, HF-631G, DFT/B3LYP/6-311+G and MP2-6-311G, respectively. In addition, the ionization potentials for TODB equal 9.123 and 6.703 eV, which are lower than the DODB values using the same basis sets semi-empirical PM6 and DFT/B3LYP/6-311+G, respectively. It is clear, from the resulting (IP) and (Δ*E*) values that TODB has the lowest values at the same calculated basis sets compared with DODB. Thus, TODB will have the higher ability to adsorb on the mild steel surface than the DODB inhibitor. Furthermore, the hardness (*η*) and softness (*σ*) related to each other and the lowest hardness compound have the highest softness value.^70^ From the mentioned results in Table 11, the softness (*σ*) values are equal to 0.2769, 0.2203, 0.6395 and 0.2244 eV^−1^ for TODB, and the DODB values are equal to 0.2501, 0.2144, 0.5757 and 0.2209 eV^−1^ using the semi-empirical PM6, HF-631G, DFT/B3LYP/6-311+G and MP2-6-311G basis sets, respectively. In addition, the hardness (*η*) results are equal to 3.611, 4.539, 1.563 and 4.457 eV for TODB, and the DODB values are equal to 3.998, 4.663, 1.737 and 4.526 eV using semi-empirical PM6, HF-631G, DFT/B3LYP/6-311+G and MP2-6-311G basis sets, respectively. From these mentioned values, TODB shows softness results higher than DODB and the opposite is detected for hardness. This indicates that the ability of TODB to protect the steel surface is greater than DODB. The transferred (Δ*N*) electrons give significant proof for the inhibition ability by electron donation from the inhibitor (Lewis base) to the surface of the metal (Lewis acid). From the resulting values in Table 11, the ability of TODB to donate electrons is higher than DODB, and the calculated values are 0.5947 and 0.5416 (e) using DFT/B3LYP/6-311+, respectively. As a result of this Δ*N* effect, a coordination bond is formed between the inhibitor and the (vacant d orbitals) metal surface.^71–73^ In addition, other parameters are shown in Table 11, including the electronegativity (*χ*), total negative charge (TNC), molecular volume (M. V.) and electrophilicity (*ω*). The theoretical values are in good agreement with the experimental values, and also suggest that TODB has higher inhibition ability than DODB.

**Table 11:** The calculated quantum chemical parameters using several optimization bases sets: semi empirical PM6, HF-631G, DFT-B3LYP/6-311G and MP2-6-311G

OPT	Molecule	*E*_HOMO_	*E*_LUMO_	Δ*E*	IP	μ	M. V.	TNC	*σ*	*ω*	*χ*	*η*	Δ*N*	*η*_PDP_
		(eV)	(eV)	(eV)	(eV)	(D)	(cm^3^ mol^−1^)	(e)	(eV^−1^)	(eV)	(eV)	(eV)	(e)	(%)
Semi empirical-PM6	TODB	−9.1237	−1.9002	7.2235	9.1237	2.8692	299.301	−6.7216	0.2769	4.2059	5.5119	3.611	0.2060	94.94
	DODB	−9.4758	−1.4792	7.9966	9.4758	8.3631	412.278	−8.8698	0.2501	3.7520	5.4775	3.998	0.1904	93.58
HF-631G	TODB	−8.8766	0.2019	9.0785	8.8766	6.4045	308.450	−7.4865	0.2203	2.0722	4.3374	4.539	0.2933	94.94
	DODB	−8.8374	0.4895	9.3270	8.8374	8.2401	252.661	−8.2896	0.2144	1.8679	4.1740	4.663	0.3030	93.58
DFT -B3LYP/6-311G	TODB	−6.7038	−3.5764	3.1274	6.703	5.0238	280.103	−5.498	0.6395	8.4481	5.1401	1.563	0.5947	94.94
	DODB	−6.8554	−3.3813	3.4741	6.855	7.3262	330.502	−6.536	0.5757	7.5408	5.1183	1.737	0.5416	93.58
MP2-6-311G	TODB	−9.1057	−0.1918	8.9139	9.1057	4.4017	240.277	−6.5932	0.2244	2.4244	4.6488	4.457	0.2638	94.94
	DODB	−8.8807	0.1717	9.0524	8.8807	9.7925	296.3680	−7.6450	0.2209	2.0947	4.3545	4.526	0.2922	93.58

### 3.10. Monte Carlo simulations

**Fig. 14:**
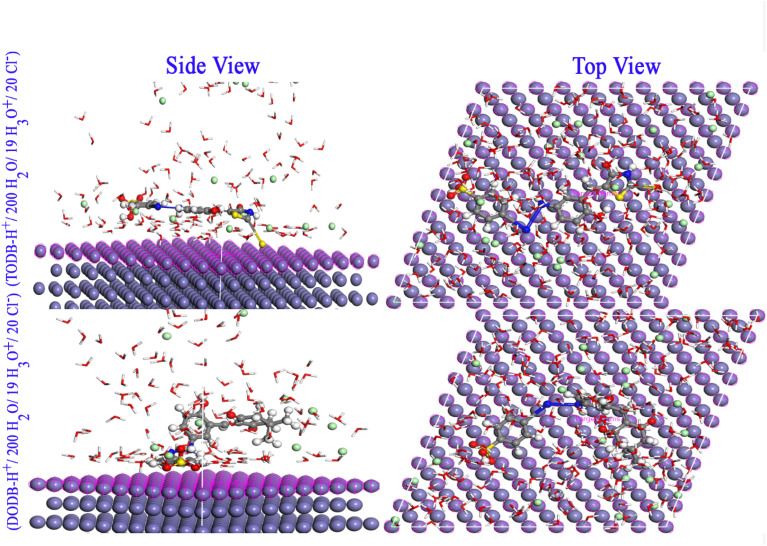
Monte Carlo simulations for the most favorable modes of adsorption obtained for TODB-H^+^ and DODB-H^+^ on Fe (1 1 0) surface, side and top view.

The adsorption of the two inhibitor molecules (either neutral TODB and DODB, or protonated TODB-H^+^ and DODB-H^+^) was simulated in vacuum and aqueous phases. Fig. 14 depicts the most stable configurations of TODB-H^+^ and DODB-H^+^ on the Fe (110) surface in the aqueous conditions. Table 12 shows the final result output descriptors of the MC simulation for the adsorption process in both vacuum and aqueous phases. The inhibitor molecules were clearly adsorbed on the Fe surface in a parallel adsorption arrangement, which guaranteed maximum surface coverage and optimum protection from corrosive particles. All adsorption energies are negative values, indicating spontaneous and stable adsorption. This finding might be related to the electron contributions from oxygen, nitrogen, sulfur, lone pairs and π electrons to the vacant d-orbitals of Fe (110), resulting in the formation of a protective coating on the metal surface. The *E*_ads_ values show that TODB is superior to the DODB molecule, which is in agreement with the DFT and the experimental findings. This can be attributed to the stronger electron-donating effect of the extra C=S, S and N–H groups in the TODB molecule, which promotes stronger adsorption on Fe (110), as compared to the (C=O) and methoxy groups in DODB.

**Table 12:** The outputs and descriptors calculated by the Monte Carlo simulations for adsorption of TODB and DODB on Fe (110) (in kcal mol^−1^)

Phase	Inhibitor	Total energy (kcal mol^−1^)	Adsorption energy (kcal mol^−1^)	Rigid adsorption energy (kcal mol^−1^)	Deformation energy (kcal mol^−1^)	(d*E*_ads/dNi_) (kcal mol^−1^)	Binding energy (kcal mol^−1^)	*η*_PDP_ (%)
Gas phase	TODB	−267.348	−3647.916	−217.758	−3430.157	−3647.916	3647.916	94.94
	DODB	−314.846	−3025.009	−219.216	−2805.793	−3025.009	3025.009	93.58
	TODB–H^+^	−279.357	−1839.355	−217.128	−1622.226	−1839.355	1839.355	94.94
	DODN-H^+^	−344.996	−1567.746	−223.388	−1344.358	−1567.746	1567.746	93.58
Aqueous phase	TODB	- 6089.077	- 9629.112	−6183.978	−3445.134	−3534.668	3534.668	94.94
	DODB	−6187.867	−9057.498	−6231.939	−2825.560	−2962.374	2962.374	93.58
	TODB–H^+^	−6129.486	−7854.973	−6215.992	−1638.982	−1869.789	1869.789	94.94
	DODB-H^+^	−6115.986	−7504.225	−6129.681	−1374.544	−1508.008	1508.008	93.58

## 4. Conclusions

From our theoretical and experimental obtained values, the following conclusions can be made:

1. Two novels azo arylidene derivatives were synthesized and characterized.

2. By increasing the temperature and the inhibitor concentration, the inhibition efficiency increases.

3. The PDP curves indicate that these derivatives behave as mixed type inhibitors.

4. The adsorption of these derivatives on the MS surface is a mixed one, *i.e.*, physical and chemical, and obeyed the Langmuir model.

5. UV, SEM and EDX measurements supported the formation of a preventive film from the inhibitors on the MS surface.

6. Gravimetric, ICPE, electrochemical measurements and theoretical quantum calculations are in agreement.

7. The bio-corrosion related to the presence of SRB can be controlled by the current azo arylidene derivatives.

## Conflicts of interest

The authors declare that they have no known competing financial interests or personal relationships that could have appeared to influence the work reported in this paper.

## Supplementary Material
